# The Health Economics of Metastatic Hormone-Sensitive and Non-Metastatic Castration-Resistant Prostate Cancer—A Systematic Literature Review with Application to the Canadian Context

**DOI:** 10.3390/curroncol29050275

**Published:** 2022-05-07

**Authors:** Ivan Yanev, Jessy Gatete, Armen G. Aprikian, Jason Robert Guertin, Alice Dragomir

**Affiliations:** 1 Centre for Outcomes Research and Evaluation, Research Institute of McGill University Health Centre, Montreal, QC H4A 3J1, Canada; yanev.ivan@mail.mcgill.ca (I.Y.); junior.gatete@mail.mcgill.ca (J.G.J.); 2 Experimental Surgery, McGill University, Montreal, QC H3A 0G4, Canada; 3 Division of Urology, Department of Surgery, McGill University, Montreal, QC H3A 0G4, Canada; armen.aprikian@mcgill.ca; 4 Département de Médecine Sociale et Préventive, Université Laval, Quebec City, QC G1V 0A6, Canada; jason.guertin@fmed.ulaval.ca; 5 Centre de Recherche du CHU de Québec-Université Laval, Quebec City, QC G1V 4G2, Canada

**Keywords:** prostate cancer, mHSPC, NM-CRPC, review, cost analysis, cost effectiveness, clinical trials, real-world data, healthcare-system perspective, societal perspective

## Abstract

**Background:** Health economic evaluations are needed to assess the impact on the healthcare system of emerging treatment patterns for advanced prostate cancer. The objective of this study is to review the scientific literature identifying cost-effectiveness and cost analyses that are assessing treatments for metastatic hormone-sensitive prostate cancer (mHSPC) and nonmetastatic castration-resistant prostate cancer (nmCRPC). **Methods**: On 29 June 2021, we searched the scientific (MEDLINE, Embase, and EBSCO) and grey literature for health economic studies targeting mHSPC and nmCRPC. We used the CHEC-extended checklist and the Welte checklist for risk-of-bias assessment and transferability analysis, respectively. **Results**: We retained 20 cost-effectiveness and 4 cost analyses in the mHSPC setting, and 14 cost-effectiveness and 6 cost analyses in the nmCRPC setting. Docetaxel in combination with androgen deprivation therapy (ADT) was the most cost-effective treatment in the mHSPC setting. Apalutamide, darolutamide, and enzalutamide presented similar results vs. ADT alone and were identified as cost-effective treatments for nmCRPC. An increase in costs as patients transitioned from nmCRPC to mCRPC was noted. **Conclusions**: We concluded that there is an important unmet need for health economic evaluations in the mHSPC and nmCRPC setting incorporating real-world data to support healthcare decision making.

## 1. Introduction

Advanced prostate cancer (PCa) is associated with poor quality of life and high mortality [1]. The health states preceding the terminal stage of PCa are metastatic hormone-sensitive prostate cancer (mHSPC) and nonmetastatic castration-resistant prostate cancer (nmCRPC). Metastatic hormone-sensitive prostate cancer is characterized by de novo metastasis while the patient is still responsive to medical or surgical castration [1]. In 2018, approximately 1200 men were diagnosed in Canada with mHSPC [2]. Nonmetastatic castration-resistant prostate cancer is characterized by rising prostate-specific antigen (PSA) levels despite castrate levels of testosterone, without metastasis being detected by conventional imaging [3]. Virtually all mHSPC and nmCRPC patients will eventually progress, develop metastasis, and present significant morbidities and paraneoplastic effects [3,4].

Targeting these health states and aiming to delay progression, the 2019 Canadian Urological Association guidelines recommend [4] the use of androgen-deprivation therapy (ADT) for newly diagnosed mHSPC patients. Docetaxel in combination with ADT (DOCE) is recommended for patients with high-volume disease and good performance status. Enzalutamide + ADT (ENZA) and apalutamide + ADT (APA) are also recommended as systemic therapy alternatives for mHSPC treatment. Abiraterone acetate + prednisone + ADT (ABI) can be considered as an option for low-volume mHSPC, but is recommended for patients presenting at least two of the following criteria: Gleason score of ≥8, presence of three or more lesions on bone scan, or presence of measurable visceral metastasis [4]. The 2020 Canadian Urological Association guidelines recommend the use of either APA, ENZA, or darolutamide + ADT (DARO) for high-risk nmCRPC patients, defined by a PSA-doubling time shorter than 10 months [3]. While these treatment options are successfully proven to delay progression and improve survival, they increase the financial burden on the healthcare system. The costs associated with novel PCa treatments are being added to the already growing burden of the disease as the incidence of PCa is increasing due to the aging population [5]. There is a need for health economic evaluations to appropriately assess the impact of these novel therapies in order to better understand the evolution of the burden associated with PCa and optimize resource allocation to improve disease management. Therefore, this systematic review is necessary to synthesize the current state of the health economic literature regarding advanced PCa.

The objective of this project is to systematically review the scientific literature identifying economic evaluation studies that are assessing the latest treatments for mHSPC and nmCRPC. Consequently, this study aims to identify potential knowledge gaps in health economic evidence for the integration of novel treatments for advanced PCa.

## 2. Materials and Methods

### 2.1. Eligibility Criteria

We built our inclusion criteria around the population of male patients that have been clinically diagnosed with mHSPC or nmCRPC. We considered all interventions that were recommended for mHSPC and nmCRPC in the Canadian Urological Association guidelines. For outcomes, we targeted costs, the burden of disease, or cost-effectiveness results that referred to Health Canada-approved treatments for mHSPC and nmCRPC, regardless of the country of origin of these studies. As treatment guidelines may differ in different jurisdictions, we did not stratify our analyses further than by health state (mHSPC or nmCRPC). Within our eligibility criteria, we considered studies using data from clinical trials as well as studies using real-world data to capture the full extent of the literature.

The inclusion criteria that were used for study selection were cost-effectiveness analysis, cost-of-illness analysis, health technology assessment (HTA), economic evaluation, and disease-burden analysis (analysis that estimates the financial impact of PCa). We excluded studies referencing only mCRPC without analyzing mHSPC or nmCRPC, other reviews, meta-analyses, and studies that did not present costs. Additionally, we excluded budget impact analyses (BIAs) because they are highly payer-specific, and they consider costs of given products, projected market shares, incidence, prevalence, and indication restrictions [6,7]. Budget impact analyses report on the affordability of a particular health technology for a specific payer based on their purchasing power, and therefore they lack transferability between payers and healthcare systems. Furthermore, BIAs contain confidential elements that are often not publicly disclosed [8].

### 2.2. Literature Search

We searched MEDLINE, Embase, EBSCO and the grey literature (National Institute for Health and Care Excellence (NICE) Evidence database) on 29 June 2021. As data collection was initiated prior to study registration, this systematic review was not eligible for registration in PROSPERO and does not have a registration number. Based on our search strategy and database verification, there is no similar registered study in PROSPERO prior to the submission date of this manuscript. Our search strategy was centered around three concepts and was reviewed by an experienced librarian. The first concept was designed to capture economic evaluations, models, and cost analyses and is based on the Canadian Agency for Drug and Technologies in Health (CADTH) search filter developed for literature reviews [9]. The second concept aimed to capture the advanced stages of PCa and was constructed by combining the medical subject heading (MeSH) terms and keywords such as “Prostatic Neoplasms”, “Neoplasm Metastasis” and “Castration-Resistant” referring to mHSPC and nmCRPC. Since mCRPC is the terminal stage of advanced PCa, we included it in the search criteria to ensure the capture of studies referencing the pre-mCRPC period. This wider search strategy allows for a thorough review of the literature and captures studies reporting on mHSPC or nmCRPC that might have been wrongfully tagged as mCRPC. The third concept represented the combination of search terms for medications and therapies that are currently approved in Canada for the treatment of advanced PCa. The full search strategy and results for MEDLINE are available in Appendix A, Table A1 and were adapted for the other databases of interest. We considered all original research publications and abstracts published in English from 2010 to the present day, to capture all relevant publications.

### 2.3. Study Selection

Search results were uploaded into Covidence [10], a web-based licensed software designed to facilitate and improve literature reviews. Duplicates were detected and removed automatically by Covidence [10]. Two reviewers (IY, JJG) independently conducted a title and abstract screening to retain pertinent articles that satisfied the inclusion criteria. Conflicts were resolved by consulting with a third independent reviewer (AD). Full-text review was then performed independently by two reviewers (IY, JJG). We rejected irrelevant studies and documented the reason for rejection. Conflicts at that stage were resolved by discussion among the two reviewers. The third reviewer (AD) was consulted when an agreement was not reached.

### 2.4. Data-Collection Process

Data items were collected by an extraction form (available in Appendix A, Table A2) that we adapted from Wijnen et al. [11] to fit our specific study objective as recommended. When multiple references reported data from the same study, only the final or most mature report was considered. Data extraction was validated by a second reviewer (JJG).

### 2.5. Data Items

When available, we extracted the following information: the reference health state, the type of analysis, the study base type (model vs. trial-based), the intervention, the comparator or the current standard of care, the perspective, the methods of cost measurement, the costs, the methods of effect measurement, the effects in life years gained (LYGs) or quality-adjusted life-years (QALYs), the incremental cost-effectiveness ratio (ICER), and the sensitivity analysis. Additionally, we sought data regarding the year of valuation, the time horizon, the discounting rate, the authors, the preferred strategy, the type of publication, the setting, and the sponsor.

### 2.6. Assessments from HTA Agencies

By reviewing the grey literature, we captured assessments of interest that contained cost-effectiveness analyses from the United Kingdom’s NICE and the Scottish Medicines Consortium (SMC). To complement this information and reflect the Canadian governmental assessment of therapies for advanced PCa, health economic analyses of the target medications were extracted from the CADTH and Institut National de l’Excellence en Santé et en Services Sociaux (INESSS) databases.

### 2.7. Risk-of-Bias Assessment

We performed a risk-of-bias assessment on the selected studies using the Consensus on Health Economic Criteria (CHEC) extended checklist [12,13] for critical appraisal of the quality of the economic evaluations (available in Appendix A, Table A3) as recommended by the Cochrane collaboration [11]. Through this questionnaire, we evaluated potential sources of bias, structural assumptions for modeling, outcome valuation, and if study conclusions were supported by their results. The CHEC extended checklist was used because of its high scrutiny and its ability to assess model-based economic evaluations [11]. We classified the studies as “Excellent”, “Good”, “Fair” and “Poor” based on their score in the risk-of-bias assessment questionnaire. This grading system, which has not been validated, considered that all the items of the questionnaire carried the same weight. The questionnaire items were judged dichotomously: 1 point was awarded if a study satisfied an item from the questionnaire; no point was awarded if item fulfillment was unclear, unspecified, or insufficient. Therefore, we quantified the quality of the studies by their total score (maximum score of 20) to be able to identify the higher-quality studies. Studies that scored 17 or higher were considered of excellent quality, 15–16 of good quality, 13–14 of fair quality, and 12 or lower of poor quality.

### 2.8. Transferability Analysis

Furthermore, we evaluated the transferability of the economic evaluations, which is the ability to hold true for different populations or settings [14] by using the Welte checklist [15]. The Welte checklist was used due to its ability to assess trial and model-based economic evaluations as well as the fact that it uses clear cut-off points to determine if a study is transferable [11]. The Welte checklist is a decision chart for assessing and improving the transferability of economic evaluation results between countries [15]. This decision chart includes knockout criteria, a checklist of transferability factors, and a component that evaluates the uncertainty of transferred results. The knockout criteria are defined by three characteristics that a study needs to satisfy for its results to be transferable to the study country, and they are used as cut-off points to determine transferability. Studies were grouped by the country-specific setting of the conducted analysis and transferability to the Canadian setting was assessed. The International Society for Pharmacoeconomics and Outcomes Research (ISPOR) Country-Specific Pharmacoeconomic Guidelines were used as a reference for evaluating the methodological characteristics [16]. Healthcare system characteristics were evaluated through the provided information within the retained references. Population characteristics were evaluated through an online search [17,18,19].

### 2.9. Effect Measures

As we extracted crude effectiveness measures in either LYGs or QALYs, we did not use any synthesis methods to report these outcomes. Additionally, we extracted costs and ICERs. In cost analyses, cost components were reported as they were reported by the original authors. When probabilistic sensitivity analyses were available, they were reported as the probability that an ICER is inferior to the prespecified willingness-to pay-threshold.

### 2.10. Synthesis Methods

No statistical analyses were performed in the reporting of costs or outcomes. All costs were converted to 2021 Canadian dollars and adjusted for inflation by using historical currency exchange rates [20] and the Canadian historical consumer price index, respectively [21]. On the rare occasions that the year of cost valuation was not reported, the year of publication was considered the year of valuation. When discounting rates were not reported, we assumed that the analysis was conducted using recommended local discounting rates. No extrapolation was performed for missing data; therefore, only data retrieved from publications were reported.

## 3. Results

### 3.1. Summary

**Figure 1:**
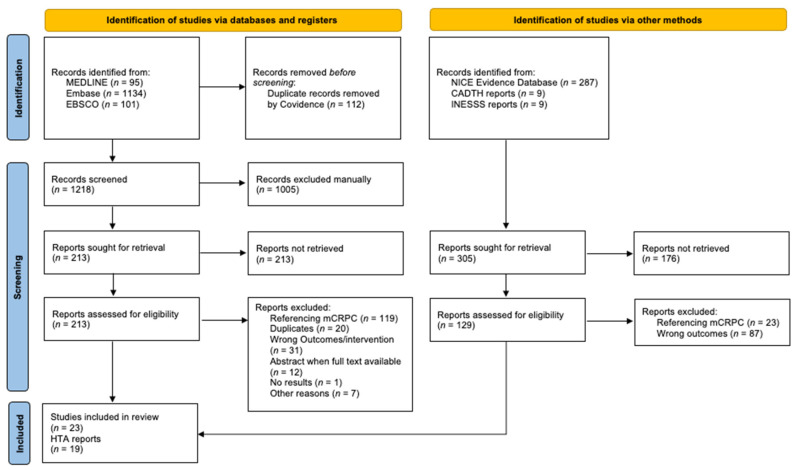
PRISMA flow diagram. Abbreviations: CADTH: Canadian Agency for Drugs and Technologies in Health, HTA: health technology assessment, INESSS: Institut national d’excellence en santé et en services sociaux, NICE: National Institute for Health and Care Excellence, mCRPC: metastatic castration-resistant prostate cancer.

Through our literature search, we captured 1330 records from our database search and 305 grey-literature records, which resulted in 1505 nonduplicate citations of original research articles, abstracts, or reports that were screened for relevance (Figure 1 based on PRISMA reporting guidelines [22]). Among those, 213 (13%) database records and 129 (7.9%) grey-literature records were retained for full-text screening. The final analysis included 23 (1.4%) database records and 19 (1.2%) grey-literature records. Of these, 24 studies referred to mHSPC and 20 to nmCRPC.

The characteristics of the retained records are available in Table 1. The predominant type of health economic evaluation was cost-effectiveness analysis with 19 [23,24,25,26,27,28,29,30,31,32,33,34,35,36,37,38,39,40,41,42] and 16 records [41,43,44,45,46,47,48,49,50,51,52,53,54,55] in mHSPC and nmCRPC, respectively. There were 4 [56,57,58,59] cost analyses referencing mHSPC and 6 [59,60,61,62,63,64] referencing nmCRPC. When analyzing the characteristics of the included publications, 10 studies were conducted in the United States [33,35,36,55,57,60,61,62,63,64], 11 in the United Kingdom [27,28,29,30,42,48,49,50,51,52], 4 in China [38,39,40,56], and 2 from Brazil [31,41]. There were only two academic studies that were conducted from a Canadian perspective [32,58]. From the retained studies, 13 used partitioned-survival analysis models [23,25,28,29,30,32,43,44,47,48,50,52,54], 12 used Markov models [24,26,34,35,36,37,38,39,40,45,55], and 4 used semi-Markov models [27,49,51,53], while only 2 used analytical models [31,41]. The healthcare-system perspective was the predominant perspective used in the captured analyses, while the societal perspective was only used by six studies [25,26,38,40,47,55]. All of the cost-effectiveness analyses referred to efficacy data from clinical trials. Only seven cost studies used real-word data to support their analysis [57,59,60,61,62,63,64].

#### 3.1.1. Assessments from HTA Agencies

Through the NICE and SMC databases, five HTA reports for mHSPC and five for nmCRPC were captured. The Canadian HTA entities (CADTH and INESSS) have published five reports for nmCRPC and four reports for mHSPC, which all contained cost-effectiveness analyses, except for one report regarding darolutamide that included a cost-minimization analysis.

#### 3.1.2. Economic Evaluations

Willingness-to-pay thresholds referred to in this paragraph are those considered by the original authors and reflect local standards. In the mHSPC setting, 11 studies evaluated DOCE and 10 of them analyzed ADT alone as an alternate option (Table 2). On the other hand, Pelloux-Prayer et al. (2020) [34] assessed treatment sequencing. They identified the sequence of DOCE, followed by ABI, as being the cost-effective option for asymptomatic and mildly symptomatic patients when compared to DOCE followed by ENZA (ICER of 708,983 CAD/QALY). In symptomatic patients, repeating DOCE compared to cabazitaxel (CABA) after the failure of DOCE was the preferred option, as the CABA sequence was associated with an excessive ICER of 1,869,295 CAD/QALY. Docetaxel was analyzed versus ABI in five studies [33,34,35,36,41], and against ENZA in two studies [34,66]. There seems to be a consensus that DOCE is the cost-effective treatment for mHSPC compared to ADT alone, with ICERs ranging from 9045 CAD/QALY to 70,459 CAD/QALY. The two studies that did not consider DOCE as cost-effective are a Chinese [40] and a Brazilian [41] study that reports ICERs exceeding the local willingness-to-pay thresholds (20,301 USD/QALY and 33,000 USD/QALY, respectively). A study by Zheng et al. (2021) [39] evaluated the cost-effectiveness of ENZA compared to ADT and rejected ENZA with ICERs of 538,940 CAD/QALY in the US perspective and 281,948 CAD/QALY in the Chinese perspective, as they exceeded local willingness-to-pay thresholds.

In the nmCRPC setting, two cost-effectiveness analyses evaluated APA in comparison to ENZA [53,54] (Table 2). The study by Tsiatas et al. identified APA as the cost-effective treatment, with an ICER ranging from 10,938–54,417 CAD/QALY from the Greek perspective. On the other hand, Toro et al. identified ENZA as the cost-effective treatment with an ICER of CAD 97,934 vs. ADT and dominated APA from the Mexican perspective. Zhou et al. (2018) analyzed the cost-effectiveness of APA vs. ADT from the Chinese perspective and observed an excessive ICER of 944,906 CAD/QALY, qualifying ADT as the preferred treatment. Aguiar et al. (2017) analyzed DOCE vs. ADT alone and observed an ICER of 36,875 CAD/QALY in favor of DOCE, which remained cost-effective in 53% of the scenarios in the probabilistic sensitivity analysis.

Regarding the HTAs conducted by governmental authorities in the mHSPC setting, ENZA, APA, DOCE, and ABI were assessed. These evaluations fall in line with the published literature, identifying DOCE as the cost-effective treatment for mHSPC when compared to the alternatives. Reported ICERs are within the acceptable range when comparing APA, ABI, and ENZA to ADT. However, comparing these novel therapies against DOCE yields high ICERs (200,000 CAD/QALY and more). These high ICERs occasionally lead to favorable recommendations for reimbursement based on the provided clinical benefit and improved quality of life. These favorable recommendations are often made conditionally to the attenuation of the financial burden through price reductions or patient access schemes.

In the nmCRPC setting, CADTH and INESSS both identify APA, DARO, and ENZA as more effective treatments compared to ADT, and are associated with ICERs ranging from CAD184,879 to 1,237,896 per QALY [24,26,46,47]. However, these treatments received positive recommendations based on their abilities to improve quality of life and delay metastases with the condition that the financial burden is reduced. The evaluations conducted for DARO vs. ADT by the SMC and ENZA vs. ADT by NICE were associated with ICERs of 31,927–47,890 CAD/QALY [50] and 24,996 CAD/QALY [49], respectively, led to favorable recommendations. These lower ICERs in comparison to the Canadian assessments are in part due to patient access schemes. In their HTA of ENZA for nmCRPC, NICE [49] concluded that ENZA in combination with ADT is not cost-effective vs. ADT alone at the provided list price. They recommended APA and DARO for reimbursement in the nmCRPC setting [48,52]. However, these treatments were associated with excessive ICERs, and NICE’s recommendations were made conditional to financial rebates provided by the manufacturers.

#### 3.1.3. Cost-Analysis Studies

Among the studies that conducted a cost analysis in the mHSPC setting, Hu et al. [56] identified that using DOCE instead of ABI would represent a cost-saving alternative in China (Table 3). Wong et al. [58] reported the cost of treating mHSPC with ABI to vary from CAD 540,299 to CAD 797,544 for a period of 42 to 44 months. Treating mHSPC patients with ENZA resulted in costs of CAD 225,387 to CAD 602,822 for a period of 12–36 months. This analysis identified the main cost factor as the duration of the mHSPC state.

Svenson et al. assessed that the cost for healthcare resource utilization in the mHSPC setting in Sweden was CAD 11,893 per year. Ke et al. assessed the cost of mHSPC per patient per year to be CAD 188,676 for the Medicare Advantage population and CAD 125,060 for the commercially insured US population.

In the nmCRPC setting, Svenson et al. concluded that the healthcare resource utilization in the nmCRPC setting would cost CAD 6024 per patient per year (Table 3). Freedland et al. [60] observed that the yearly cost per patient increased from CAD 5121 to CAD 16,014 after the onset of nmCRPC in the US. Shah et al. [63] assessed the increase in cost due to adverse events in nmCRPC that reached CAD 63,619 compared to CAD 47,212 per patient without adverse events. Central nervous system adverse events were an important cost driver. Four studies analyzed the cost increase as patients transitioned from nmCRPC to mCRPC [60,61,62,64]. George et al. [61] reported an increase in PCa-related costs from CAD 556 to CAD 3675 and all-cause medical costs that increased from CAD 1883 to CAD 5460 for nmCRPC and mCRPC, respectively. Wu et al. [64] reported an increase in the medical and pharmacy costs within the Medigap and commercially insured patients. Medicare Advantage and Medigap are both supplementary private insurance plans that beneficiaries can opt for. They differ in the fact that Medigap policies are neither provided nor endorsed by the United States Government, while Medicare Advantage plans are provided by government-approved private companies [67,68].

#### 3.1.4. Results from Real-World Data Studies

This review captured seven studies using real-world data to conduct health economic evaluations. There were two publications assessing the mHSPC setting and 5 assessing the nmCRPC setting. Additionally, it is important to mention that all these studies were cost analyses. Furthermore, none of the studies using real-world data conducted a direct comparison between treatments. Instead, these studies focused on reporting the financial impact caused by various elements. Shah et al. [63] reported the increase in costs due to adverse events while others evaluated cost differences due to the transition from nmCRPC to mCRPC [60,61,62,64]. All the real-world studies, with the exception of the study by Svensson et al., were conducted in the United States and used the Veterans’ Health Administration (VHA) database or private insurance databases. The study by Svensson et al., on the other hand, was conducted in Sweden [59].

#### 3.1.5. Risk-of-Bias Assessment

Results from the risk-of-bias assessment are reported in Table 4. We classified 12 studies as excellent, 6 as good, 2 as fair, and 3 as poor. Issues relating to generalizability, ethics, and distribution were the predominant sources of bias.

#### 3.1.6. Transferability Assessment

Studies were grouped by country of origin of the conducted analysis. Transferability of economic studies from Brazil, China, Columbia, France, Greece, Mexico, Sweden, the United Kingdom, and the United States to the Canadian setting was evaluated (Appendix A, Table A4). The correspondence between study country and Canada is summarized in Table 5. General knockout criteria were respected throughout all of the countries of interest [15]. When focussing on the methodological characteristics of the analyses, all the reference countries present unbiased or slight underestimates [16]. This is due to the use of higher discount rates, as current discounting in Canada is fixed at 1.5% by the CADTH guidelines [69]. Additionally, the studies do not consider the cost of productivity loss from a societal perspective. When analyzing the healthcare-system characteristics, technology availability was consistent across all the studied countries. Price variability and absolute and relative prices of healthcare, however, seem to be important sources of bias affecting the transferability to the Canadian setting. Population characteristics of Greece, France, Sweden, and the United Kingdom presented high correspondence to the Canadian ones [15]. However, Brazil, China, Columbia, Mexico, and the United States presented a few differences that might bias the transferability of the studies and yield lower ICERs. Transferring results from studies conducted in these countries is therefore subject to a potential bias that may lead to an underestimation. Therefore, these results should be transferred with caution considering this greater uncertainty. Disease incidence and prevalence, life expectancy, work-loss time, health-status preference, and productivity are potentially sources of transferability bias that could over- or underestimate results leading to erroneous conclusions [15,17,18,19].

## 4. Discussion

### 4.1. Summary of Results

The emergence of novel treatments for advanced PCa led to an improvement in survival and quality of life. Given the high costs of these medications, health economic evaluations are needed to maximize the clinical benefit for patients while controlling the financial burden on the healthcare system. The Canadian setting was used as a reference throughout the manuscript, given the fact that Canada has a robust health-technology assessment process that is extensive, well-referenced, expert-reviewed, and used as a benchmark for HTA worldwide. Through this project, we reviewed the scientific and grey literature for health economic studies targeting the latest treatments for mHSPC and nmCRPC approved by Health Canada, analyzed their potential benefit in the management of PCa in Canada, and identified knowledge gaps. This systematic literature review identified 24 and 20 health economic studies in the health states of mHSPC and nmCRPC, respectively, with the predominant type of analysis being cost-effectiveness analysis. The risk-of-bias assessment confirmed that the retrieved studies are of good quality in general. While only a few academic studies were conducted from a Canadian perspective, transferability analysis suggested that results from foreign studies would incorporate a small to medium level of bias if interpreted in the Canadian setting.

Our study identified 142 references, 80 of which included cost-effectiveness analyses for mCRPC, that were excluded from our analysis. Relative to the well-established health economic literature in mCRPC, the health economic literature for mHSPC and nmCRPC is still immature and there is a need for increased efforts to provide evidence-based support to healthcare decision-making. There is a significant unmet need for health economic evaluations that target mHSPC and nmCRPC and carry through disease progression until death while integrating all active treatment options and that are adapted to the Canadian setting.

### 4.2. mHSPC

The current literature review demonstrated that DOCE in combination with ADT was determined to be the most cost-effective treatment in the mHSPC setting. Compared to DOCE, comparators such as ENZA, APA, and ABI yield ICERs that are exceeding the predefined willingness-to-pay thresholds due to small incremental effectiveness benefits that are outweighed by considerably higher costs. This was also underlined in the cost analysis by Hu et al. 2019 [56], where the costs associated with ABI were 3 times greater than the costs of DOCE, CAD 259,909 vs. CAD 80,754 in the healthcare system perspective and CAD 64,510 vs. CAD 18,823 in the patient perspective. Hu et al. 2019 [56] ranked as a study of good quality according to our risk-of-bias assessment, but its results might be an underestimation of the costs according to our transferability analysis. It is important to mention that manufacturers often provide rebates to improve these ICERs. In Canada, the pan-Canadian Pharmaceutical Alliance (pCPA) is an organization comprised of provincial, territorial, and federal governments that aims to increase the value of publicly funded drug programs through their combined negotiating power [70]. Joint negotiations led by the pCPA for the reimbursement of ABI, APA, DARO, and ENZA for mHSPC and nmCRPC [71] have led to listing agreements and private discounted prices for these medications. Furthermore, patent expirations give birth to generic products that are available at lower prices. As of 2021, generic versions of abiraterone acetate are available on the Canadian market, some of which cost 73% less than the brand name product [72]. These lower prices will undoubtedly have an important impact, potentially making ABI the cost-effective option, as the price of abiraterone acetate was identified to have a major impact on the ICER [31,33,35,36,56]. Given the general trend, quality, and relatively good transferability of the retrieved studies, we can conclude that DOCE is the cost-effective treatment for mHSPC. These results could potentially be reversed if cost rebates on new acquisition prices are considered.

### 4.3. nmCRPC

In the nmCRPC setting, the results from this literature review inform that APA, DARO, and ENZA are considered cost-effective when compared to ADT alone. Furthermore, these three medications have similar ICERs compared to ADT alone, because they have demonstrated similar efficacy in clinical trials [73,74,75] and have similar drug acquisition prices in Canada [76]. It would be relevant to conduct a cost-effectiveness analysis with real-world data to compare their effectiveness in the Canadian setting. A Japanese real-world evidence analysis studied ENZA’s effectiveness through a long-term medical records review [77]. In this study, Fujiwara et al. reported similar overall survival and slightly inferior progression-free survival when benchmarking against the PROSPER, PREVAIL, and AFFIRM clinical trials [77], which can be an indication that the effectiveness of ENZA would yield similar cost-effectiveness results if conducted with real-world data.

Cost analyses show an increase in healthcare costs as patients progress to metastatic disease underlining the importance of delaying progression. This increase is perceived in the inpatient and outpatient settings by Seal et al. [62] and Freedland et al. [60], where inpatient costs can be increased by up to threefold per patient per year after the appearance of metastasis. This increase can be perceived in the medical, pharmaceutical, inpatient, and outpatient costs [60,62,64].

### 4.4. Real-World Data Studies

As this review captured only a few health economic studies (i.e., cost analyses) using real-world data, it appears that clinical trials remain the main data source for conducting cost-effectiveness analysis in the nmCRPC and mHSPC settings. Real-world data represented the data source of choice for cost analysis, where researchers were able to determine the financial impact of transition between health states or the increased costs of treatment due to adverse events. The use of real-world data from administrative databases allows researchers to capture larger sample sizes, has greater external validity, and is more representative of clinical practice as patients outside of clinical trials tend to be older and have more comorbidities relative to trial patients.

### 4.5. Risk-of-Bias Assessment

The risk-of-bias assessment demonstrated that the selected studies were of good quality with a few exceptions. In general, studies did not satisfy the following criteria of the checklist: assumptions, costs measure methods, generalizability, and ethical and distributional issues. This underreporting can be explained by a lack of consideration or by the fact the authors conscientiously omitted the specification to comply with publication-specific constraints. This is an important aspect to acknowledge, since certain records are conference abstracts. In those cases, it would be impossible to report the full extent of the scientific effort. The CHEC extended checklist was selected for the risk-of-bias assessment as it is proven to be of greater scrutiny than others and it is recommended by the Cochrane collaboration [11]. Furthermore, the CHEC extended checklist is not only suitable for assessing modeling analysis but also cost analysis, which was one of its main advantages over the ISPOR questionnaire to assess relevance and credibility by Caro et al. [78] The Philips checklist [79] was another suitable option; however, because of its numerous criteria, it is not recommended for use in the assessment of a large number of studies.

### 4.6. Strengths

To our knowledge, this is the first systematic review that combines the health economic evaluations of mHSPC and nmCRPC. Furthermore, this study is the only one that considers governmental reports while conducting transferability analysis to the Canadian perspective. Through our literature review, we have encountered a similar review conducted by Grochtdreis et al. in 2018 [80], where the authors searched for cost-effectiveness analyses and cost-of-illness analyses targeting treatment for the CRPC and mCRPC. Quality assessment was conducted by using the CHEERS checklist and the risk of bias was assessed by the Bias in the Economic Evaluations checklist [80]. While this study was of great methodological quality, it did not consider the grey literature or analyses from HTA agencies, and nor did it conduct a transferability analysis. Furthermore, Grochtdreis et al. [80] did not extend their search to the mHSPC health state.

Through our review, we identified significant knowledge gaps. For instance, very few studies consider mHSPC and nmCRPC simultaneously in their analysis, the primary reason being that these are mutually exclusive health states that require a specific indication for a drug to be used. That being said, there is an important androgen receptor-axis-targeted therapies (ARAT) usage overlap in the mHSPC and the nmCRPC settings. Moreover, as both health states eventually lead to mCRPC, considering them jointly integrates a more complete spectrum of the disease. Additionally, as cost-effectiveness analysis is often used to justify treatment reimbursement, analyses were designed to compare active adjunct treatment plus ADT to ADT alone. Given the growing landscape of treatments for advanced PCa, future health economic models should not only consider ADT as the standard of care but also consider the other active treatments that are given in combination with ADT, as was conducted in CADTH’s pharmacoeconomic report of APA for mHSPC [23]. In this study, APA was benchmarked against DOCE, ABI, and ADT alone. Furthermore, some studies are conducted from the societal perspective that may be biased as they do not provide indirect costing components such as productivity loss to the patient and the healthcare provider. However, patient productivity loss is likely to be low, given that PCa is a disease of old age with the average age of diagnosis being above 65 [81]. Nonetheless, this should be acknowledged in the design and discussed by the authors as it is an important part of the societal perspective.

### 4.7. Limitations

This systematic review was based on peer-reviewed methods designed specifically for health economic articles and was conducted with great scrutiny [11]. However, as with all systemic reviews, this study has certain limitations. Because the number of captured studies was relatively low and because they did not always report results by subgroup of patients based on disease severity, we could not stratify our analyses beyond the health states of mHSPC and nmCRPC. As this review protocol was not registered in PROSPERO, it was not peer-reviewed and may incorporate a certain level of bias. To overcome this bias, the review protocol was designed to have wide inclusion criteria and cover various databases, including the grey literature. By reviewing the grey literature, conference abstracts, and reports that are not peer-reviewed, the research exposes itself to biases. Correctly assessing the quality of these publications is not possible as some of these publications are not reporting their full protocols and results, either due to publication-length limits or confidentiality agreements. To tackle this problem, other literature reviews have excluded conference abstracts and governmental HTAs [80]. We decided to include grey literature in our analysis to preserve a high level of sensitivity in our analysis. We were, however, faced with a challenge when assessing the risk of bias in abstracts and governmental reports. For abstracts, we considered that all unreported items from the CHEC extended checklist were omitted and therefore might have underestimated the quality of some publications. While we considered all the items of the CHEC extended checklist to carry the same weight, this grading scheme has not been validated. It is important to mention that the criteria list of the CHEC extended checklist is regarded as a minimum standard [13]. A good-quality health economic study should therefore satisfy all the items. Consequently, the CHEC extended checklist is not intended to be used as a grading system and these results should be interpreted with caution. Through our analysis, we did not capture a single study that satisfied all the items, and only five publications had one unsatisfactory item. This indicates that there is an unmet need for high-quality publications in the field.

We decided to exclude governmental HTA reports from the risk-of-bias assessment analysis because of the high level of underreporting due to confidentiality agreements. Furthermore, HTA reports from CADTH and INESSS were not captured by our search and were added manually to satisfy the scope of this analysis. This could potentially lead to article-selection bias or the omission of certain reports. It is important to mention that HTA entities do not provide sufficient information for model reconstruction and model validation by peer scientists because of confidentiality agreements with treatment manufacturers. However, their results remain important for consideration, serving as a robust benchmark for academic research. Ignoring them will lead to a significant study-selection bias.

Another limitation of this study is that we were not able to integrate cost-effectiveness thresholds in the analysis because they are country- or healthcare-system-specific. The United Kingdom’s NICE uses an official explicit cost-effectiveness threshold of GBP 20,000 to GBP 30,000 per QALY. In the United States, this threshold is between USD 50,000 to USD 100,000, while in Canada the same threshold is being referred to, but in Canadian dollars. Although the United States and Canada have historically referred to these thresholds without officially endorsing them; certain medications exceeding these thresholds have been judged cost-effective. Furthermore, converting these thresholds from their local currency to 2021 CAD may result in significant bias and is not considered a recommended practice as they have not been updated to reflect the current country-specific purchasing power. We have decided therefore not to benchmark our results against these thresholds that are not always explicitly endorsed and that might be biased as they have not been updated to reflect the current value of money and country-specific purchasing power.

## 5. Conclusions

This literature review describes the current state of health economic studies on mHSPC and nmCRPC. We identified docetaxel plus ADT to be the cost-effective treatment for mHSPC in most of the retained publications. Enzalutamide, apalutamide, and darolutamide—all in addition to ADT—were associated with similar ICERs when compared to ADT alone. Additionally, through the risk-of-bias assessment and transferability analyses we found that while the current literature provides guidance, study results cannot be applied directly to the Canadian healthcare system without incorporating a certain degree of bias. Finally, we conclude that the scientific literature is immature. We identify an important unmet need for health economic evaluations in the mHSPC and nmCRPC settings incorporating Canadian real-world data to support healthcare decision-making to effectively manage advanced PCa.

## Figures and Tables

**Table 1:** Characteristics of retained economic studies.

Type of Evaluation	Country	Year	First Author	Health State	Treatment and Comparator	Data Source	Model Type	Perspective	Year of Value
CA	Canada	2020	Wong [58]	mHSPC	ABI vs. ENZA	ATITUDE, STAMPEDE, ENZAMET, and ARCHES	-	NR	2017
CA	China	2019	Hu [56]	mHSPC	ABI vs. DOCE vs. ADT	CHAARTED, LATITUDE and GETUG-AFU-15	-	Healthcare system and patient	2017
CA	Sweden	2021	Svenson [59]	mHSPC, nmCRPC	-	Real-world data PCa data Base Sweden	-	Healthcare system	2018
CA	US	2014	Seal [62]	nmCRPC	-	Real-world data Patients in the Premier Perspective Database	-	Institutional	2010
CA	US	2018	George [61]	nmCRPC	-	Real-world data Veterans’ Health Administration (VHA) database	-	Healthcare system	NR
CA	US	2019	Ke [57]	mHSPC	-	Real-world data (Optum Clinformatics Extended DataMart)	-	Public and private payer	2018
CA	US	2020	Shah [63]	nmCRPC	ENZA vs. ABI vs. bicalutamide	Real-world data MarketScan database	-	Private payer	2017
CA	US	2020	Wu [64]	nmCRPC	-	Real-world data Truven Health MarketScan Commercial and Medicare Supplemental (Medigap) databases	-	Public and private payer	2016
CA	US	2020	Freedland [60]	nmCRPC	-	Real-world data Veterans Health Administration (VHA) database	-	Healthcare system	2016
CE	Brazil	2017	Aguiar [41]	mHSPC, nmCRPC	ABI vs. DOCE vs. ADT	GETUG-AFU 15 and CHAARTED	Analytical model	Public payer	2016
CE	Brazil	2019	Aguiar [31]	mHSPC	ABI vs. DOCE vs. ADT	STAMPEDE	Descriptive analytic model	Public payer	2017
CE	Canada	2018	CADTH 4 [44]	nmCRPC	APA vs. ADT	SPARTAN	Partitioned-survival model	Government	2018
CE	Canada	2018	INESSS 3 [47]	nmCRPC	APA vs. ADT DOCE	SPARTAN	Partitioned-survival model	Healthcare system/Societal	2018
CE	Canada	2019	Beca [32]	mHSPC	DOCE vs. ADT	CHAARTED	Partitioned-survival model	Public payer	2017
CE	Canada	2019	CADTH 3 [45]	nmCRPC	ENZA vs. ADT APA	PROSPER, SPARTAN	Markov model	Healthcare payer	2018
CE	Canada	2020	CADTH 1 [23]	mHSPC	APA vs. ADT vs. DOCE vs. ABI	TITAN	Partitioned-survival model	Public payer	2020
CE	Canada	2020	CADTH 2 [24]	mHSPC	ENZA vs. ADT vs. DOCE vs. APA vs. ABI	ARCHES and ENZAMET	Markov model	Public payer	2020
CE	Canada	2020	INESSS 1 [26]	mHSPC	ENZA vs. ADT vs. DOCE	ARCHES, ENZAMET, and MAenR	Markov model	Societal	2020
CE	Canada	2020	INESSS 2 [25]	mHSPC	APA vs. ADT	SPARTAN	Partitioned-survival model	Societal	2020
CE	Canada	2020	CADTH 5 [43]	nmCRPC	DARO vs. ADT	ARAMIS	Partitioned-survival model	Public payer	2018
CE	China	2017	Zheng [40]	mHSPC	DOCE vs. ADT	CHAARTED	Markov model	Societal	2015
CE	China	2017	Zhang [38]	mHSPC	Za vs. DOCE vs. DOCE+Za vs. ADT	Clinical trials	Markov model	Societal	2016
CE	France	2021	Pelloux-Prayer [34]	mHSPC	DOCE vs. ABI vs. ENZA vs. caba sequencing	CHAARTED, LATITUDE, COU-AA-302, PREVAIL, FIRSTANA	Markov model	Healthcare system	2020
CE	Greece	2019	Tsiatas [54]	nmCRPC	APA vs. ENZA	SPARTAN and PROSPER	Partitioned-survival model	Healthcare system	NR
CE	Mexico	2020	Toro [53]	nmCRPC	ENZA vs. APA vs. ADT	Clinical Trials	Semi-Markov model	Public payer	2018
CE	UK	2016	NICE 2 [42]	mHSPC	DOCE vs. ADT	STAMPEDE, CHAARETED, GETUG-AFU 15	-	Healthcare system	2015
CE	UK	2018	Woods [37]	mHSPC	DOCE vs. ADT	STAMPEDE	Markov model	Healthcare system	2014
CE	UK	2019	NICE 5 [49]	nmCRPC	ENZA vs. ADT	PROSPER	Semi-Markov partitioned-survival model	Healthcare system	2018
CE	UK	2019	Scottish Medicines 2 [51]	nmCRPC	ENZA vs. ABI	PROSPER	Semi-Markov model	Healthcare system	2019
CE	UK	2020	Scottish Medicines 1 [27]	mHSPC	ABI vs. ADT DOCE	LATITUDE	Semi-Markov/Partitioned-survival	Healthcare system	2019
CE	UK	2020	NICE 7 [48]	nmCRPC	DARO vs. ADT	ARAMIS	Partitioned-survival model	Healthcare system	2020
CE	UK	2020	Scottish Medicines 3 [50]	nmCRPC	DARO vs. ADT	ARAMIS	Partitioned-survival model	Healthcare system	2020
CE	UK	2021	NICE 1 [28]	mHSPC	ENZA vs. ADT	ARCHES	Partitioned-survival model	Healthcare system	2020
CE	UK	2021	NICE 3 [29]	mHSPC	ABI vs. ADT vs DOCE	LATITUDE, STAMPEDE	Partitioned-survival model	Healthcare system	2021
CE	UK	2021	NICE 4 [30]	mHSPC	APA vs. ADT	TITAN	Partitioned-survival model	Healthcare system	2021
CE	UK	2021	NICE 6 [52]	nmCRPC	APA vs. ADT	SPARTAN	Partitioned-survival model	Healthcare system	2021
CE	US	2018	Zhou [55]	nmCRPC	APA vs. ADT	SPARTAN	Markov model	Societal	NR
CE	US	2019	Ramamurthy [35]	mHSPC	ABI vs. DOCE vs. ADT	CHAARTED, LATITUDE	Markov model	Public payer	2018
CE	US	2019	Sathianathen [36]	mHSPC	ABI vs. DOCE vs. ADT	GETUG-AFU15, CHAARTED, LATITUDE	Markov model	Private payer	2017
CE	US	2020	Parikh [33]	mHSPC	MDT vs. ABI followed by DOCE vs. DOCE followed ABI	STOMP, STAMPEDE, TAX-327, COU-AA-301	Markov model	Public payer	2020
CE	US/China	2021	Zhang [65]	mHSPC	ENZA vs. ADT	Clinical Trials	Markov model	Public payer	NR
CE/cost-minimization	Canada	2020	INESSS 4 [46]	nmCRPC	DARO vs. APA	ARAMIS	-	Healthcare system	2020

ARAMIS, ARCEHS, ENZAMET, CHAARTED, COU-AA-302, FIRSTANA, GETUG-AFU 15, LATITUDE, MAenR, PREVAIL, PROSPER, STAMPEDE, STOMP, SPARTAN, TAX-327, and TITAN are registered randomized clinical trials. Abbreviations: ABI: abiraterone acetate + prednisone + ADT, ADT: androgen-deprivation therapy, APA; Apalutamide + ADT, CA: cost analysis, Caba: cabazitaxel, CE: cost-effectiveness, DARO: darolutamide + ADT, DOCE: docetaxel + ADT, ENZA: enzalutamide + ADT, MDT: metastasis-directed therapy, mHSPC: metastatic hormone-sensitive prostate cancer, nmCRPC: nonmetastatic castration-resistant prostate cancer, NR: Not reported.

**Table 2:** Costs, ICERs, and probability of cost effectiveness for CEA in mHSPC and nmCRPC.

First Author	Disc. Rate	Effectiveness	Cost	Cost Effectiveness (ICER)	Sensitivity Analysis	Cost-Effective Strategy Based on Specific Local WTP Thresholds
mHSPC						
Zheng [40]	3%	DOCE: 1.85 QALY ADT: 1.26 QALY	DOCE: CAD 38,520 ADT: CAD 20,293	37,973 CAD/QALY	PA demonstrated that when WTP threshold was lower than CAD 57,740 ADT alone was cost-effective.	ADT
Ramamurthy [35]	None	ADT: 1.21 PF-QALY DOCE: 1.53 PF-QALY ABI: 1.73 PF-QALY	ADT: CAD 14,444 DOCE: CAD 36,912 ABI: CAD 315,648	DOCE: 70,459 CAD/QALY ABI: 1,409,461 CAD/QALY	PA: In 99.5% of scenarios, DOCE is cost-effective with a WTP of 209,331 CAD/PF-QALY.	DOCE
Parikh [33]	3%	MDT: 4.63 QALY ABI: 4.89 QALY ADT: 4.00 QALY	MDT: CAD 197,394 ABI->DOCE: CAD 233,278 DOCE+ABI: CAD 190,410	MDT: CAD 450,649 NMB ABI->DOCE: CAD 450,339 NMB DOCE->ABI: CAD 368,372 NMB	PA: 53.6% of simulations MDT was the cost-effective strategy	MDT
Beca [32]	1.5%	DOCE: 3.915QALY ADT: 2.852 QALY	DOCE: CAD 147,427 ADT: CAD 119,287	25,478 CAD/QALY	1WSA yield ICERs below 36,809 CAD/QALY	DOCE
Zhang 2021 [39]	China: 3% US: 3.%	US: ADT: 4.09 QALY ENZA: 6.21 QALY China: ADT: 3.78 QALY ENZA: 5.70 QALY	US: ADT: CAD 604,365 ENZA:CAD 1,746,917 China: ADT: CAD 104,624 ENZA: CAD 645,965	US: 538,940 CAD/QALY China: 281,948 CAD/QALY	1WSA demonstrated the utility for the PFS state and the cost of ENZA were the most influential	ADT
Woods [37]	3.5%	ADT: 4.90 QALY DOCE: 5.79 QALY	nm: ADT: CAD 90,409 DOCE: CAD 89,998 mets: ADT: CAD 86,066 DOCE: CAD 90,637	nm: DOCE: Dominant mets: DOCE: 9,045 CAD/QALY	Price of DOCE was sensitive to increase ICER above the 21,325 CAD/QALY threshold.	DOC
Zhang 2017 [38]	3%	ADT: 2.65 QALY Za+ADT: 2.69 QALY DOCE: 2.85 QALY DOCE+Za: 2.78 QALY	ADT: CAD 29,820 Za+ADT: CAD 35,554 DOCE: CAD 40,905 DOCE+Za: CAD 46,417	ADT: CAD 29,820; 2.65 QALY Za+ADT: CAD 35,554; 2.69 QALY; 143,351 CAD/QALY DOCE+ADT: CAD 40,905; 2.85 QALY; 55,429 CAD/QALY DOCE+Za+ADT: CAD 46,417; 2.78QALY; 127,679 CAD/QALY	1WSA: The most impactful parameter were failure-free survival (FFS) state, cost of ADT, and utility of FFS state. PA confirmed conclusions, however SOC alone was the cost-effective option at a WTP threshold of CAD 28,870.	DOCE
Sathianathen [36]	3%	ADT: 2.435 QALY DOCE: 2.737 QALY ABI: 4.272 QALY	ADT: CAD 286,885 DOCE: CAD 301,516 ABI: CAD 933,864	DOCE: 48,457 CAD/QALY ABI: 411,980 CAD/QALY	ABI represented value high-health care only one threshold exceeded CAD 488,439.	DOCE
Aguiar 2019 [31]	NR	ABI vs. ADT: 0.999 QALY gain DOCE vs. ADT: 0.492 QALY gain	ABI vs. ADT: CAD 164,826 DOCE vs. ADT: CAD 62,517		With an incremental investment of CAD 49,522 DOCE is cost-effective treatment in 91% of cases.	ADT at Brazilian threshold DOCE at WHO threshold
Aguiar 2017 [41]	NR	HR nm: 0.12 QALY benefit of DOCE Metastatic: 0.52 QALY benefit of DOCE	DOCE: CAD 28,149 ADT: CAD 19,554	Metastatic: 15,968 CAD/QALY HV metastatic disease: 11,970 CAD/QALY	Metastatic: 80% of scenarios DOCE cost-effective HV metastatic disease: 73% of scenarios DOCE cost-effective	DOCE
Pelloux-Prayer [34]	2.5%	Asymptomatic/mildly symptomatic: DOCE->ABI: 4.24 LY DOCE->ENZA: 4.25 LY ABI->DOCE: 3.97 LY ABI->ENZA: 4.15 LY Symptomatic: DOCE->DOCE: 4.05 LY DOCE->Caba: 4.07 LY ABI->DOCE: 3.97 LY	Asymptomatic/mildly symptomatic: DOCE->ABI: CAD 144,133 DOCE->ENZA: CAD 285,649 ABI->DOCE: CAD 222,858 ABI->ENZA: CAD 250,395 Symptomatic: DOCE->DOCE: CAD 121,140 DOCE->Caba: CAD 157,253 ABI->DOCE: CAD 222,858	Asymptomatic/mildly symptomatic: DOCE-> ENZA vs. DOCE->ABI = 708,983 CAD/QALY (ABI->DOCE, ABI->ENZA is dominated) Symptomatic: DOCE->Caba vs. DOCE->DOCE= 1,869,295 CAD/QALY (ABI->DOCE is dominated)	Asymptomatic/mildly symptomatic: Cost reduction of 70% of ABI or ENZA led to ABI->ENZA to become efficient at the 74,353 CAD/LY threshold. Symptomatic: Cost reduction of 70% of ABI and Caba leads to ABI->DOCE to be least costly and effective but ICER for the two other options exceeds the cost-effectiveness threshold.	DOCE
CADTH 1 [23]	1.5%	NR	NR	ADT<980 CAD/QALY DOCE between 980 and 294,494 CAD/QALY; ABI is the preferred option if the WTP is more than 294,494 CAD/QALY.	NR	DOCE
CADTH 2 [24]	1.5%	ENZA vs. DOCE 0.24 QALY	ENZA vs. DOCE: CAD 75,566	ENZA vs. DOCE: 307,776 CAD/QALY	<=52,200 CAD/QALY = 0% need 75% price reduction	DOCE
INESSS 1 [26]	1.5%	ENZA: 1.24 QALY ADT:0.13 QALY	ENZA vs. ADT CAD 152,469 (CAD 152,571–172,193) ENZA vs. DOCE CAD 122,906 (CAD 123,015–128,428)	vs ADT 122,755 CAD/QALY vs. DOCE 924,765 CAD/QALY	ENZA vs. ADT 107,253–138,837 CAD/QALY ENZA vs. DOCE 662,362–1,438,466 CAD/QALY	DOCE
INESSS 2 [25]	1.5%	APA vs. ADT: 1.45QALY	APA vs. ADT: CAD 138,070.00	APA vs. ADT: 95,484 CAD/QALY	86,471–113,580 CAD/QALY <=52,200 CAD/QALY = 4% <=104,400 CAD/QALY = 57%	APA
NICE 1 [28]	3.5%	NR	NR	NR	NR	ENZA
NICE 2 [42]	3.5%	OS benefit of 10–15 months	Cost of 6 cycles of DOCE: CAD 10,018	NR	NR	NR
NICE 3 [29]	3.5%	NR	NR	>148,706 CAD/QALY gained vs. DOCE >44,612 CAD/QALY vs. ADT	NR	ABI is not recommended
NICE 4 [30]	3.5%	NR	NR	Acceptable ICER would be lower than the middle of the range 29,741 to 44,227 CAD/QALY	NR	APA is recommended only if: DOCE is not suitable and the price of APA is rebated
Scottish Medicines 1 [27]	3.5%	ABI vs. ADT: 0.987 ABI vs. DOCE: 0.401		ABI vs. ADT: CAD 144,442 ABI vs. DOCE: CAD 321,706	ABI vs. ADT: CAD 103,527–167,146 ABI vs. DOCE: CAD 254,536–515,315	NR
nmCRPC						
Aguiar 2017 [41]		DOCE vs. ADT: 0.12 QALY	DOCE vs. ADT: CAD 4424	DOCE vs. ADT: 36,875 CAD/QALY	In PA, 53% of the scenarios evaluated were cost-effective based on the three-fold gross domestic product (GDP) per capita 46,929 CAD/QALY.	DOCE
Zhou [55]	NR	APA:NR ADT: NR	APA:NR ADT: NR	Apa vs. ADT ACER: 223,720 CAD/QALY ICER: 944,906 CAD/QALY	1WSA demonstrated that OS and costs have the greatest impact on the results.	ADT
Tsiatas [54]	Yes	APA: 4.3 QALY ENZA: 3.8 QALY	APA: CAD 205,951 to 228,558 ENZA: CAD 200,263	CAD 10,938 to 54,417	APA cost-effective in 56% to 68% of scenarios at WTP threshold of CAD 78,154	APA
Toro [53]	5%	ENZA: 3.75 QALY APA: 3.27 QALY ADT: 3.00 QALY	ENZA: CAD 78,348 APA: CAD 91,406 ADT: CAD 765	ENZA vs. ADT: 97,934.84 CAD/QALY Enza vs. APA: dominating	None	ENZA
CADTH 3 [45]	1.5%	ENZA vs. ADT:0.44 ENZA vs. Apa+ADT: −0.28	ADT: CAD 106,081 APA: CAD −6158	ENZA vs. ADT: 243,679 CAD/QALY APA: 25,666 CAD/QALY *	NR	ENZA
CADTH 4 [44]	1.5%	APA vs. ADT: 0.57 QALY	APA vs. ADT: CAD 12,1193	213,176 CAD/QALY	NR	APA
CADTH 5 [43]	1.5%	DARO vs. ADT: 0.78 QALY	DARO vs. ADT: CAD 144,504	DARO vs. ADT: 184,879 CAD/QALY	NR	DARO
INESSS 3 [47]	1.5%	APA vs. ADT: 0.05	APA vs. ADT: CAD 67,692	APA vs. ADT: 1,237,896 CAD/QALY	146,975–10,032,238 CAD/QALY	APA
INESSS 4 [46] *	1.5%	NR	DARO vs. ADT: CAD 3551 (same as APA)	NR	NR	DARO
NICE 5 [49]	3.5%	NR	NR	ENZA vs. ADT: 92,138 CAD/QALY	NR	ENZA is not cost-efficient vs. ADT
NICE 6 [52]	3.5%	NR	NR	NR	Middle of the range normally considered a cost-effective use of NHS resources	APA
NICE 7 [48]	3.5%	Survival in mCRPC 3–4 shorter after DARO than ADT	NR	NR	31,927–47,890 CAD/QALY	DARO
Scottish Medicines 2 [51]	3.5%	ADT: 3.18 ENZA: 4.17	ADT: CAD 122,016 ENZA: CAD 271,587	ENZA vs. ADT: 150,857 CAD/QALY with PAS	109,921–431,601 CAD/QALY	ENZA is not cost-efficient
Scottish Medicines 3 [50]	3.5%	NR	NR	NR	NR	DARO

All costs are reported in 2021 CAD. Abbreviations: ABI: abiraterone acetate + prednisone, ACER: average cost-effectiveness ratio, ADT: androgen-deprivation therapy, APA; apalutamide, Caba: cabazitaxel, DOCE: docetaxel + ADT, DARO: darolutamide + ADT, ENZA: enzalutamide + ADT, GDP: gross domestic product, HV: high volume, MDT: metastasis-directed therapy, PF-QALY: progression-free quality-adjusted life year, PFS: progression-free survival, PPPY: per patient per year, PA: probabilistic sensitivity analysis, SD: standard deviation, SOC: standard of care, QALY: quality-adjusted life year, WHO: World Health Organization, WTP: willingness to pay, Za: zoledronic acid, 1WSA: one-way sensitivity analysis. * INESSS 4 presents the results of a cost-minimization analysis.

**Table 3:** Costs, ICERs, and probability of cost effectiveness for CEA in mHSPC and nmCRPC.

First Author	Time Period of Reported Costs	Costing Methods	Inpatient Costs	Outpatient Cost	Medical Costs	Pharmaceutical Costs	Cancer Specific Costs	Total Costs
mHSPC								
Hu [56]	Lifetime	Decision-analytic model						
Healthcare perspective	-	-	-	-	DOCE: CAD 5877 ABI: CAD 6329	DOCE: CAD 26,432 ABI CAD 248,609		DOCE: CAD 80,754 ABI: CAD 259,909
Patient perspective	-	-	-	-	DOCE: CAD 1304 ABI: CAD 1582	DOCE: CAD 3802 ABI: CAD 13,029		DOCE: CAD 18,823 ABI: CAD 64,510
Wong [58]		Total prices of treatment under the trial’s experimental and control arms						
ABI (AWP)	33 to 42 months		-	-	-	-	-	CAD 540,299 to CAD 707,544
ENZA (AWP)	13 to 36 months		-	-	-	-	-	CAD 225,387 to CAD 602,822
Svensson [59]	12 months	Bottom-up	-	-	-	-	-	CAD 11,893.00
Ke [57]	1 year	Top-down						
U.S. Medicare Advantage	-		CAD 188,676	-	-	-	-	-
Commercially-insured	-		CAD 174,525	-	-	-	-	-
nmCRPC								
Shaha [63]	1 year	Bottom-up						
CNS AEs			-	-	-	-	-	AEs: CAD 71,485 No AE: CAD 45,582
Any AEs			-	-	-	-	-	AEs: CAD 63,619 No AE: CAD 47,212
Seal [62]	Mean cost per patient	Top-down						
nmCRPC			CAD 15,062	CAD 5576	-	-	-	CAD 9338
mCRPC			CAD 17,837	CAD 8680	-	-	-	CAD 12,267
Wu [64]		Top-down						
Commercial	nmCRPC: 12.0 months mCRPC: 13.9 months		-	-	nmCRPC: CAD 36,452 mCRPC: CAD 108,741	nmCRPC: CAD 4373 mCRPC: CAD 8180	-	nmCRPC: CAD 40,825 mCRPC: CAD 254,743
Medigap	nmCRPC: 12.0 months mCRPC: 14.6 months		-	-	nmCRPC: CAD 31,976 mCRPC: CAD 72,686	nmCRPC: CAD 6,551 mCRPC: CAD 101,651	-	nmCRPC: CAD 38,527 mCRPC: CAD 195,547
Svensson [59]	12 months	Bottom-up	-	-	-	-	-	CAD 6024
George [61]	4 years until death, health plan disenrollment or the study end date	Top-down						
nmCRPC			-	-	CAD 1883		CAD 556	-
mCRPC			-	-	CAD 5460		CAD 3675	-
Freedland [60] *	1 year	Top-down						
nmCRPC			CAD 5121	CAD 13,803	-	CAD 2900	-	-
mCRPC			CAD 16,014	CAD 19,559	-	CAD 9564	-	-

All costs are reported in 2021 CAD. Abbreviations: ABI: abiraterone acetate + prednisone + ADT, ADT: androgen-deprivation therapy, AE: adverse events, CNS: central nervous system, DOCE: docetaxel + ADT, ENZA: enzalutamide + ADT; ICER: incremental cost-effectiveness ratio, nmCRPC: nonmetastatic castration-resistance prostate cancer, mCRPC: metastatic castration-resistance prostate cancer, PC: prostate cancer, PPPY: per patient per year, SD: standard deviation, WTP: willingness to pay. * Freedland et al. report additional emergency costs of CAD 508 and CAD 947 per year for nmCRPC and Mcrpc, respectively.

**Table 4:** Quality assessment of selected mHSPC and nmCRPC studies.

	Questionnaire Item																				
Study ID	Population	Competing alternatives	Research question	Design	Assumptions/validation	Time horizon	Perspective	Costs identification	Costs measure	Costs valuation	Outcome identification	Outcome measure	Outcome valuation	Incremental analysis	Discounting	Sensitivity analysis	Conclusions	Generalizability	Conflict of interest	Ethical/distributional	Total
mHSPC																					
Pelloux-Prayer [34]																					19
Sathianathen [36]																					19
Zhang 2017 [38]																					19
Zhang 2021 [39]																					19
Woods [37]																					19
Parikh [33]																					18
Zheng [40]																					18
Beca [32]																					17
Aguiar 2019 [31]																					17
Hu [56]																					16
Ramamurthy [35]																					16
Svensson [59]																					15
Ke [57]																					11
Wong [58]																					11
Aguiar 2017 [41] *																					18
nmCRPC																					
Toro [53]																					17
Freedland [60]																					17
Shah [63]																					16
Zhou [55]																					16
Wu [64]																					16
Seal [62]																					14
Tsiatas [54]																					14
George [61]																					11

* [41] report results both for mHSPC and nmCRPC. Green indicates that the article satisfied the item. Red indicates that the item was now satisfied or not reported.

**Table 5:** Correspondence between study country and Canada.

	Brazil	China	Columbia	France	Greece	Mexico	Sweden	UK	US
Methodological Characteristics									
Perspective	Medium (societal vs. public payer)	Very high	Medium	High	Medium (societal vs. healthcare)	High	High	High	High (payer/societal)
Discount rate	Low (not reported)	Medium (1.5% vs. 3%)	Low (not reported)	High (1.5% vs. 2.5%)	Low (not reported)	Low (1.5% vs. 5%)	Low	Medium (1.5% vs. 3.5%)	Medium (1.5 vs. 3%)
Medical cost approach	Low (AE not considered)	High	Medium	High	Low (not described)	High	High	High	High
Productivity cost approach	Low (not considered)	High	Low (not reported)	Low	Low (not considered)	Low (not considered)	Low (not measured)	Low (not evaluated)	Low (not evaluated)
Healthcare-System Characteristics									
Absolute and relative prices in health care	Medium	High	Medium	Medium	Medium	Medium	Medium	Medium	Medium
Practice variation	Medium	Medium	Medium	Medium	Medium	Medium	High	Medium	High
Technology availability	High	High	High	Very high	High	High	High	Very high	High
Population characteristics									
Disease incidence/prevalence	Medium	Low	Medium	Very high	High	High	High	Very high	Medium
Case-mix	Medium	Low	Medium	High	High	Medium	High	High	Medium
Life expectancy	Medium	Medium (80 vs. 75)	Medium	Very high	High	Medium	High	Very high	Medium (80.0 vs. 76.3)
Health-status preferences	High	Very high	High	Very high	Medium	Medium	High	Very high	High
Acceptance, compliance, and incentives to patients	Medium	Medium	High	High	Medium	Medium	High	High	High
Productivity and work-loss time	Low (not considered)	Medium	Low (not reported)	High	Low (not considered)	Low (not considered)	Low (not measured)	High	Low (not measured)

## Data Availability

Not applicable.

## Appendix A

**Table A1:** Search strategy for Embase (searched on Thursday, 22 July 2021 8:20:33 p.m.).

Embase <1996 to 2021 Week 28>		
#	Query	Results
1	((hormone or castrat *) adj (sensitive or naive) adj prostat * adj25 (metasta * or oligometasta * or oligo-metasta * or micrometasta * or micro-metasta *)).tw.	944
2	(mHSPC or m-HSPC or mHNPC or m-HNPC or mCSPC or m-CSPC or mCNPC or m-CNPC).tw.	527
3	1 or 2	1042
4	Animal/not (Animal/and Human/)	699,130
5	3 not 4	1042
6	Castration resistant prostate cancer/and (nonmetastatic or non-metastatic).tw.	633
7	(castrat * adj (resistant or independent) adj prostat * adj25 (nonmetastatic or non-metastatic)).tw.	517
8	((androgen or hormone) adj (independent or insensitive or resistant or refractory) adj prostat * adj25 (nonmetastatic or non-metastatic)).tw.	12
9	(nmCRPC or nm-CRPC).tw.	293
10	6 or 7 or 8 or 9	728
11	Animal/not (Animal/and Human/)	699,130
12	10 not 11	728
13	Castration resistant prostate cancer/and exp metastasis/	5668
14	Castration resistant prostate cancer/and (metasta* or oligometasta * or oligo-metasta * or micrometasta * or micro-metasta *).tw.	9287
15	Castration resistant prostate cancer/and ((cancer or tumor? or tumour? or neoplasm?) adj1 (spread * or disseminat * or migration? or seeding? or circulating)).tw.	897
16	(mCRPC or m-CRPC).tw.	5538
17	(castrat * adj (resistant or independent) adj prostat * adj25 (metasta * or oligometasta * or oligo-metasta * or micrometasta * or micro-metasta *)).tw.	8775
18	(castrat * adj (resistant or independent) adj prostat * adj25 ((cancer or tumor? or tumour? or neoplasm?) adj1 (spread* or disseminat * or migration? or seeding? or circulating))).tw.	441
19	((androgen or hormone) adj (independent or insensitive or resistant or refractory) adj prostat * adj25 (metasta * or oligometasta * or oligo-metasta * or micrometasta * or micro-metasta*)).tw.	1005
20	((androgen or hormone) adj (independent or insensitive or resistant or refractory) adj prostat * adj25 ((cancer or tumor? or tumour? or neoplasm?) adj1 (spread * or disseminat* or migration? or seeding? or circulating))).tw.	11
21	13 or 14 or 15 or 16 or 17 or 18 or 19 or 20	13,816
22	Animal/not (Animal/and Human/)	699,130
23	exp docetaxel/or (docetaxel or “RP-56976” or “RP 56976” or RP56976 or RP56976s or “NSC 628503” or “NSC-628503” or NSC628503 or docetaxol or Taxoltere or Taxotere or daxotel or dexotel or docefrez or “lit 976” or “lit-976” or lit976 or oncodocel or taxespira or taxoter or texot).tw,ot.	64,427
24	abiraterone acetate/or exp abiraterone/or (abiraterone or zytiga or “154229-18-2” or “cb 7630” or “cb-7630” or cb7630 or “CB 7598” or “CB-7598” or CB7598 or yonsa).tw,ot.	8079
25	exp enzalutamide/or (enzalutamide or “MDV-3100” or MDV3100 or xtandi).tw,ot.	7708
26	exp apalutamide/or (Apalutamide or erleada or “ARN-509” or “ARN 509” or ARN509).tw,ot.	979
27	exp darolutamide/or (Darolutamide or Nubeqa or “ORM-16497” or “ORM 16497” or ORM16497 or “ODM-201” or “ODM 201” or ODM201 or “ORM-16555” or “ORM 16555” or ORM16555 or “bay 1841788” or “bay-1841788” or bay1841788).tw,ot.	435
28	exp cabazitaxel/or (cabazitaxel or kabazitaxel or Jevtana or “rpr 116258 a” or “rpr-116258-a” or “rpr 116258a” or “rpr-116258a” or rpr116258a or “txd 258” or “txd-258” or txd258 or “xrp 6258” or “xrp-6258” or xrp6258).tw,ot.	3408
29	ZOLEDRONIC ACID/or (zoledronic * or zoledronat * or zometa * or zomera * or aclasta * or zoldron * or reclast * or aredia * or m05BA08 or “CGP-42446” or “CGP 42446” or CGP42446 * or “zol-446” or “zol 446” or zol446 or “158859-43-9” or 70hz18ph24 or orazol).tw,ot.	18,442
30	(Denosumab or Xgeva or “AMG 162” or “AMG-162” or AMG162 or Prolia or amgiva).tw,ot.	6649
31	exp radium chloride ra 223/or (Ra223 or “Ra 223” or “Ra-223” or Radium223 or “Radium 223” or “Radium-223” or 223radium or “223-radium” or “223 radium” or alpharadin or xofigo or “bay 88 8223” or “bay 88-8223” or “bay88 8223” or “bay88-8223”).tw,ot.	2410
32	(Olaparib or Lymparza or “AZD-2281” or “AZD 2281” or “MK-7339” or “MK 7339 OR KU0059436”).tw,ot.	3936
33	socioeconomics/or exp “Quality of Life”/or nottingham health profile/or sickness impact profile/or exp health status indicator/or patient satisfaction/or patient preference/or daily life activity/or personal autonomy/or self concept/or sickness impact profile/	948,945
34	21 not 22	13,813
35	23 or 24 or 25 or 26 or 27 or 28 or 29 or 30 or 31 or 32	97,781
36	33 and 34 and 35	917
37	limit 36 to (human and english language and yr = “2010 -Current”)	817
38	Economics/or “cost benefit analysis”/or exp Health economics/or Budget/or exp statistical model/or Probability/or monte carlo method/or Decision Theory/or Decision Tree/or budget/or markov chain/or Cost minimization analysis/	1,250,421
39	Economics/or exp “Costs and Cost Analysis”/or Economics, Nursing/or Economics, Medical/or Economics, Pharmaceutical/or exp Economics, Hospital/or Economics, Dental/or exp “Fees and Charges”/or exp Budgets/or exp models, economic/or markov chains/or monte carlo method/or exp Decision Theory/	945,271
40	(budget * or economic * or cost or costs or costly or costing or price? or pricing or pharmacoeconomic * or pharmaco-economic * or expenditure? or expense? or financ * or (value? adj2 (money or monetary)) or Markov or monte carlo or (decision * adj2 (tree * or analy * or model *))).tw,kw.	1,296,893
41	38 or 39 or 40	2,096,764
42	34 and 35 and 41	1194
43	limit 42 to (human and english language and yr = “2010 -Current”)	1134
44	from 37 keep 1-817	817
45	((hormone or castrat *) adj (sensitive or naive) adj prostat * adj25 (metasta * or oligometasta * or oligo-metasta * or micrometasta * or micro-metasta *)).tw.	944
46	(mHSPC or m-HSPC or mHNPC or m-HNPC or mCSPC or m-CSPC or mCNPC or m-CNPC).tw.	527
47	45 or 46	1042
48	Animal/not (Animal/and Human/)	699,130
49	47 not 48	1042
50	Castration resistant prostate cancer/and (nonmetastatic or non-metastatic).tw.	633
51	(castrat * adj (resistant or independent) adj prostat * adj25 (nonmetastatic or non-metastatic)).tw.	517
52	((androgen or hormone) adj (independent or insensitive or resistant or refractory) adj prostat * adj25 (nonmetastatic or non-metastatic)).tw.	12
53	(nmCRPC or nm-CRPC).tw.	293
54	50 or 51 or 52 or 53	728
55	Animal/not (Animal/and Human/)	699,130
56	54 not 55	728
57	Castration resistant prostate cancer/and exp metastasis/	5668
58	Castration resistant prostate cancer/and (metasta * or oligometasta * or oligo-metasta * or micrometasta * or micro-metasta *).tw.	9287
59	Castration resistant prostate cancer/and ((cancer or tumor? or tumour? or neoplasm?) adj1 (spread * or disseminat * or migration? or seeding? or circulating)).tw.	897
60	(mCRPC or m-CRPC).tw.	5538
61	(castrat * adj (resistant or independent) adj prostat * adj25 (metasta * or oligometasta * or oligo-metasta* or micrometasta * or micro-metasta *)).tw.	8775
62	(castrat * adj (resistant or independent) adj prostat * adj25 ((cancer or tumor? or tumour? or neoplasm?) adj1 (spread * or disseminat * or migration? or seeding? or circulating))).tw.	441
63	((androgen or hormone) adj (independent or insensitive or resistant or refractory) adj prostat * adj25 (metasta * or oligometasta * or oligo-metasta * or micrometasta * or micro-metasta *)).tw.	1005
64	((androgen or hormone) adj (independent or insensitive or resistant or refractory) adj prostat * adj25 ((cancer or tumor? or tumour? or neoplasm?) adj1 (spread * or disseminat * or migration? or seeding? or circulating))).tw.	11
65	57 or 58 or 59 or 60 or 61 or 62 or 63 or 64	13,816
66	Animal/not (Animal/and Human/)	699,130
67	exp docetaxel/or (docetaxel or “RP-56976” or “RP 56976” or RP56976 or RP56976s or “NSC 628503” or “NSC-628503” or NSC628503 or docetaxol or Taxoltere or Taxotere or daxotel or dexotel or docefrez or “lit 976” or “lit-976” or lit976 or oncodocel or taxespira or taxoter or texot).tw,ot.	64,427
68	abiraterone acetate/or exp abiraterone/or (abiraterone or zytiga or “154229-18-2” or “cb 7630” or “cb-7630” or cb7630 or “CB 7598” or “CB-7598” or CB7598 or yonsa).tw,ot.	8079
69	exp enzalutamide/or (enzalutamide or “MDV-3100” or MDV3100 or xtandi).tw,ot.	7708
70	exp apalutamide/or (Apalutamide or erleada or “ARN-509” or “ARN 509” or ARN509).tw,ot.	979
71	exp darolutamide/or (Darolutamide or Nubeqa or “ORM-16497” or “ORM 16497” or ORM16497 or “ODM-201” or “ODM 201” or ODM201 or “ORM-16555” or “ORM 16555” or ORM16555 or “bay 1841788” or “bay-1841788” or bay1841788).tw,ot.	435
72	exp cabazitaxel/or (cabazitaxel or kabazitaxel or Jevtana or “rpr 116258 a” or “rpr-116258-a” or “rpr 116258a” or “rpr-116258a” or rpr116258a or “txd 258” or “txd-258” or txd258 or “xrp 6258” or “xrp-6258” or xrp6258).tw,ot.	3408
73	ZOLEDRONIC ACID/or (zoledronic * or zoledronat * or zometa * or zomera * or aclasta * or zoldron * or reclast * or aredia * or m05BA08 or “CGP-42446” or “CGP 42446” or CGP42446 * or “zol-446” or “zol 446” or zol446 or “158859-43-9” or 70hz18ph24 or orazol).tw,ot.	18,442
74	(Denosumab or Xgeva or “AMG 162” or “AMG-162” or AMG162 or Prolia or amgiva).tw,ot.	6649
75	exp radium chloride ra 223/or (Ra223 or “Ra 223” or “Ra-223” or Radium223 or “Radium 223” or “Radium-223” or 223radium or “223-radium” or “223 radium” or alpharadin or xofigo or “bay 88 8223” or “bay 88-8223” or “bay88 8223” or “bay88-8223”).tw,ot.	2410
76	(Olaparib or Lymparza or “AZD-2281” or “AZD 2281” or “MK-7339” or “MK 7339 OR KU0059436”).tw,ot.	3936
77	socioeconomics/or exp “Quality of Life”/or nottingham health profile/or sickness impact profile/or exp health status indicator/or patient satisfaction/or patient preference/or daily life activity/or personal autonomy/or self concept/or sickness impact profile/	948,945
78	65 not 66	13,813
79	67 or 68 or 69 or 70 or 71 or 72 or 73 or 74 or 75 or 76	97,781
80	77 and 78 and 79	917
81	limit 80 to (human and english language and yr = “2010 -Current”)	817
82	Economics/or “cost benefit analysis”/or exp Health economics/or Budget/or exp statistical model/or Probability/or monte carlo method/or Decision Theory/or Decision Tree/or budget/or markov chain/or Cost minimization analysis/	1,250,421
83	Economics/or exp “Costs and Cost Analysis”/or Economics, Nursing/or Economics, Medical/or Economics, Pharmaceutical/or exp Economics, Hospital/or Economics, Dental/or exp “Fees and Charges”/or exp Budgets/or exp models, economic/or markov chains/or monte carlo method/or exp Decision Theory/	945,271
84	(budget * or economic * or cost or costs or costly or costing or price? or pricing or pharmacoeconomic * or pharmaco-economic * or expenditure? or expense? or financ * or (value? adj2 (money or monetary)) or Markov or monte carlo or (decision * adj2 (tree * or analy * or model *))).tw,kw.	1,296,893
85	82 or 83 or 84	2,096,764
86	78 and 79 and 85	1194
87	limit 86 to (human and english language and yr = “2010–Current”)	1134

**Table A2:** Extraction Form.

Extraction Performed by:	
ID	
Author	
Year	
Publication type	
Setting	
Health state	
N (sample size)	
Type of analysis	
Trial- or model- based EE	
Intervention	
Comparator	
Outcome measure(s)	
Perspective	
Data source	
Disc. Rate	
Sponsor	
Methods of measurement of costs	
Costs	
Methods of measurement of effects	
Effects	
RESULTS (ICER/ICUR)	
Sensitivity analysis	
Favorable strategy	
Conclusions	

Abbreviations: EE: economic evaluation, ICER: incremental cost-effectiveness ratio, ICUR: incremental cost-utility ratio.

**Table A3:** Quality assessment form CHEC extended checklist [13].

Study ID	
Author	
1 Is the study population clearly described?	0/1
2 Are competing alternatives clearly described?	0/1
3 Is a well-defined research question posed in answerable form?	0/1
4 Is the economic study design appropriate to the stated objective?	0/1
5 Are the structural assumptions and the validation methods of the model properly reported?	0/1
6 Is the chosen time horizon appropriate in order to include relevant costs and consequences?	0/1
7 Is the actual perspective chosen appropriate?	0/1
8 Are all important and relevant costs for each alternative identified?	0/1
9 Are all costs measured appropriately in physical units?	0/1
10 Are costs valued appropriately?	0/1
11 Are all important and relevant outcomes for each alternative identified?	0/1
12 Are all outcomes measured appropriately?	0/1
13 Are outcomes valued appropriately?	0/1
14 Is an appropriate incremental analysis of costs and outcomes of alternatives performed?	0/1
15 Are all future costs and outcomes discounted appropriately?	0/1
16 Are all important variables, whose values are uncertain, appropriately subjected to sensitivity analysis?	0/1
17 Do the conclusions follow from the data reported?	0/1
18 Does the study discuss the generalizability of the results to other settings and patient/client groups?	0/1
19 Does the article/report indicate that there is no potential conflict of interest of study researcher(s) and funder(s)?	0/1
20 Are ethical and distributional issues discussed appropriately?	0/1
Total	/20

**Table A4:** Transferability assessment tables.

US	Estimated Relevance	Correspondence between Study A and Decision Country B	ICER of Decision (Canada) Based on ICER of Study Country (US):
General knockout criteria			
1. The evaluated technology is not comparable to the one that shall be used in the decision country.	-	NA	Passed
2. The comparator is not comparable to the one that is relevant to the decision country.	-	NA	Passed
3. The study does not possess an acceptable quality.	-	NA	Passed
Methodological characteristics			
Perspective	Very High	High (payer/societal)	Unbiased
Discount rate	Very High	Medium (1.5 vs. 3%)	Too low
Medical cost approach	Very High	High	Unbiased
Productivity cost approach	Low	Low (unreported)	Too low or too high
Healthcare-system characteristics			
Absolute and relative prices in healthcare	Very High	Medium	Too high
Practice variation	High	High	Unbiased
Technology availability	High	High	Unbiased
Population characteristics			
Disease incidence/prevalence	Very High	Medium	Too low or too high
Case-mix	High	Medium	Too low
Life expectancy	High	Medium (80.0 vs. 76.3)	Too low
Health-status preferences	High	High	Unbiased
Acceptance, compliance, and incentives to patients	Medium	High	Unbiased
Productivity and work-loss time	Low	Low (unreported)	Too low or too high
Disease spread	Not relevant (no infectious disease)		Unbiased
CHINA	Estimated relevance	Correspondence between study A and decision country B	ICER of decision Canada based on ICER of study country (China):
General knockout criteria			
1. The evaluated technology is not comparable to the one that shall be used in the decision country.	-	NA	Passed
2. The comparator is not comparable to the one that is relevant to the decision country.	-	NA	Passed
3. The study does not possess an acceptable quality.	-	NA	Passed
Methodological characteristics			
Perspective	Very High	Very high	Unbiased
Discount rate	Very High	Medium (1.5% vs. 3%)	Too low
Medical cost approach	Very High	High	Unbiased
Productivity cost approach	Low	High	Unbiased
Healthcare-system characteristics			
Absolute and relative prices in healthcare	Very High	High	Unbiased
Practice variation	High	Medium	Too low or too high
Technology availability	High	High	Unbiased
Population characteristics			
Disease incidence/prevalence	Very High	Low	Too low
Case-mix	High	Low	Too low or too high
Life expectancy	High	Medium (80 vs. 75)	Too low
Health-status preferences	High	Very high	Unbiased
Acceptance, compliance, and incentives to patients	Medium	Medium	Too low
Productivity and work-loss time	Low	Medium	Too low
Disease spread	Not relevant (no infectious disease)		Unbiased
UK	Estimated relevance	Correspondence between study A and decision country B	ICER of decision Canada based on ICER of study country (UK):
General knockout criteria			
1. The evaluated technology is not comparable to the one that shall be used in the decision country.	-	NA	Passed
2. The comparator is not comparable to the one that is relevant to the decision country.	-	NA	Passed
3. The study does not possess an acceptable quality.	-	NA	Passed
Methodological characteristics			
Perspective	Very High	High	Unbiased
Discount rate	Very High	Medium (1.5% vs. 3.5%)	Too low
Medical cost approach	Very High	High	Unbiased
Productivity cost approach	Low	Low (not evaluated)	Too low
Healthcare-system characteristics			
Absolute and relative prices in healthcare	Very High	Medium	Too high
Practice variation	High	Medium	Too high
Technology availability	High	Very high	Unbiased
Population characteristics			
Disease incidence/prevalence	Very High	Very high	Unbiased
Case-mix	High	High	Unbiased
Life expectancy	High	Very high	Unbiased
Health-status preferences	High	Very high	Unbiased
Acceptance, compliance, and incentives to patients	Medium	High	Unbiased
Productivity and work-loss time	Low	High	Unbiased
Disease spread	Not relevant (no infectious disease)		Unbiased
Brazil	Estimated relevance	Correspondence between study A and decision country B	ICER of decision (Canada) based on ICER of study country (Brazil):
General knockout criteria			
1. The evaluated technology is not comparable to the one that shall be used in the decision country.	-	NA	Passed
2. The comparator is not comparable to the one that is relevant to the decision country.	-	NA	Passed
3. The study does not possess an acceptable quality.	-	NA	Passed
Methodological characteristics			
Perspective	Very High	Medium (societal vs. public payer)	Too low
Discount rate	Very High	Low (not reported)	Too low
Medical cost approach	Very High	Low (AE not considered)	Too high
Productivity cost approach	Low	Low (not considered)	Too high
Healthcare-system characteristics			
Absolute and relative prices in healthcare	Very High	Medium	Too high
Practice variation	High	Medium	Too low or too high
Technology availability	High	High	Unbiased
Population characteristics			
Disease incidence/prevalence	Very High	Medium	Too low or too high
Case-mix	High	Medium	Too low or too high
Life expectancy	High	Medium	Too low or too high
Health-status preferences	High	High	Unbiased
Acceptance, compliance, and incentives to patients	Medium	Medium	Too low or too high
Productivity and work-loss time	Low	Low (not considered)	Too high
Disease spread	Not relevant (no infectious disease)		Unbiased
France	Estimated relevance	Correspondence between study A and decision country B	ICER of decision Canada based on ICER of study country (France):
General knockout criteria			
1. The evaluated technology is not comparable to the one that shall be used in the decision country.	-	NA	Passed
2. The comparator is not comparable to the one that is relevant to the decision country.	-	NA	Passed
3. The study does not possess an acceptable quality.	-	NA	Passed
Methodological characteristics			
Perspective	Very High	High	Unbiased
Discount rate	Very High	High (1.5% vs. 2.5%)	Too low
Medical cost approach	Very High	High	Unbiased
Productivity cost approach	Low	Low	Too low
Healthcare-system characteristics			
Absolute and relative prices in healthcare	Very High	Medium	Too low
Practice variation	High	Medium	Too low or too high
Technology availability	High	Very high	Unbiased
Population characteristics			
Disease incidence/prevalence	Very High	Very high	Unbiased
Case-mix	High	High	Unbiased
Life expectancy	High	Very high	Unbiased
Health-status preferences	High	Very high	Unbiased
Acceptance, compliance, and incentives to patients	Medium	High	Unbiased
Productivity and work-loss time	Low	High	Unbiased
Disease spread	Not relevant (no infectious disease)		Unbiased
Greece	Estimated relevance	Correspondence between study A and decision country B	ICER of decision Canada based on ICER of study country (Greece):
General knockout criteria			
1. The evaluated technology is not comparable to the one that shall be used in the decision country.	-	NA	Passed
2. The comparator is not comparable to the one that is relevant to the decision country.	-	NA	Passed
3. The study does not possess an acceptable quality.	-	NA	Passed
Methodological characteristics			
Perspective	Very High	Medium	Too low
Discount rate	Very High	Low (not reported)	Too low
Medical cost approach	Very High	Low (not described)	Too high
Productivity cost approach	Low	Low (not considered)	Too high
Healthcare-system characteristics			
Absolute and relative prices in healthcare	Very High	Medium	Too low
Practice variation	High	Medium	Too low or too high
Technology availability	High	High	Unbiased
Population characteristics			
Disease incidence/prevalence	Very High	High	Unbiased
Case-mix	High	High	Unbiased
Life expectancy	High	High	Unbiased
Health-status preferences	High	Medium	Too low or too high
Acceptance, compliance, and incentives to patients	Medium	Medium	Too low or too high
Productivity and work-loss time	Low	Low (not considered)	Too high
Disease spread	Not relevant (no infectious disease)		Unbiased
Sweden	Estimated relevance	Correspondence between study A and decision country B	ICER of decision Canada based on ICER of study country (Sweden):
General knockout criteria			
1. The evaluated technology is not comparable to the one that shall be used in the decision country.	-	NA	Passed
2. The comparator is not comparable to the one that is relevant to the decision country.	-	NA	Passed
3. The study does not possess an acceptable quality.	-	NA	Passed
Methodological characteristics			
Perspective	Very High	High	Unbiased
Discount rate	Very High	Low	Too high
Medical cost approach	Very High	High	Unbiased
Productivity cost approach	Low	Low (not measured)	Too low
Healthcare-system characteristics			
Absolute and relative prices in healthcare	Very High	Medium	Too high
Practice variation	High	High	Unbiased
Technology availability	High	High	Unbiased
Population characteristics			
Disease incidence/prevalence	Very High	High	Unbiased
Case-mix	High	High	Unbiased
Life expectancy	High	High	Unbiased
Health-status preferences	High	High	Unbiased
Acceptance, compliance, and incentives to patients	Medium	High	Unbiased
Productivity and work-loss time	Low	Low (not measured)	Too low
Disease spread	Not relevant (no infectious disease)		Unbiased
Mexico	Estimated relevance	Correspondence between study A and decision country B	ICER of decision Canada based on ICER of study country (Mexico):
General knockout criteria			
1. The evaluated technology is not comparable to the one that shall be used in the decision country.	-	NA	Passed
2. The comparator is not comparable to the one that is relevant to the decision country.	-	NA	Passed
3. The study does not possess an acceptable quality.	-	NA	Passed
Methodological characteristics			
Perspective	Very High	High	Unbiased
Discount rate	Very High	Low (1.5% vs. 5%)	Low
Medical cost approach	Very High	High	Unbiased
Productivity cost approach	Low	Low (not considered)	Too low
Healthcare-system characteristics			
Absolute and relative prices in healthcare	Very High	Medium	Too low or too high
Practice variation	High	Medium	Too low or too high
Technology availability	High	High	Unbiased
Population characteristics			
Disease incidence/prevalence	Very High	High	Unbiased
Case-mix	High	Medium	Too low or too high
Life expectancy	High	Medium	Too low
Health-status preferences	High	Medium	Too low or too high
Acceptance, compliance, and incentives to patients	Medium	Medium	Too low or too high
Productivity and work-loss time	Low	Low (not considered)	Too low
Disease spread	Not relevant (no infectious disease)		Unbiased
Columbia	Estimated relevance	Correspondence between study A and decision country B	ICER of decision Canada based on ICER of study country (Columbia)
General knockout criteria			
1. The evaluated technology is not comparable to the one that shall be used in the decision country.	-	NA	Passed
2. The comparator is not comparable to the one that is relevant to the decision country.	-	NA	Passed
3. The study does not possess an acceptable quality.	-	NA	Passed
Methodological characteristics			
Perspective	Very High	Medium	Too low
Discount rate	Very High	Low (not reported)	Too low
Medical cost approach	Very High	Medium	Too low
Productivity cost approach	Low	Low (not reported)	Too low
Healthcare-system characteristics			
Absolute and relative prices in healthcare	Very High	Medium	Too high
Practice variation	High	Medium	Too low or too high
Technology availability	High	High	Unbiased
Population characteristics			
Disease incidence/prevalence	Very High	Medium	Too low
Case-mix	High	Medium	Too low
Life expectancy	High	Medium	Too low
Health-status preferences	High	High	Unbiased
Acceptance, compliance, and incentives to patients	Medium	High	Unbiased
Productivity and work-loss time	Low	Low (not reported)	Too low
Disease spread	Not relevant (no infectious disease)		Unbiased

## References

[1] Cattrini C., Castro E., Lozano R., Zanardi E., Rubagotti A., Boccardo F., Olmos D. (2019). Current Treatment Options for Metastatic Hormone-Sensitive Prostate Cancer. Cancers.

[2] Canadian Cancer Statistics Advisory Committee (2019). Canadian Cancer Statistics 2019.

[3] Saad F., Aprikian A., Finelli A., Fleshner N.E., Gleave M., Kapoor A., Niazi T., North S.A., Pouliot F., Rendon R.A. (2021). 2021 Canadian Urological Association (CUA)—Canadian Uro Oncology Group (CUOG) Guideline: Management of Castration-Resistant Prostate Cancer (CRPC). Can. Urol. Assoc. J..

[4] So A.I., Chi K.N., Danielson B., Fleshner N.E., Kapoor A., Niazi T., Pouliot F., Rendon R.A., Shayegan B., Sridhar S. (2019). Canadian Urological Association—Canadian Urologic Oncology Group guideline on metastatic castration-naive and castration-sensitive prostate cancer. Can. Urol. Assoc. J..

[5] Grover S.A. (1997). Economics of prostate cancer: A computer model. Can. J. Urol..

[6] Mauskopf J.A., Sullivan S.D., Annemans L., Caro J., Mullins C.D., Nuijten M., Orlewska E., Watkins J., Trueman P. (2007). Principles of good practice for budget impact analysis: Report of the ISPOR Task Force on good research practices—Budget impact analysis. Value Health.

[7] Budget Impact Analysis. https://www.herc.research.va.gov/include/page.asp?id=budget-impact-analysis.

[8] Institut National D’excellence en Santé et en Services Sociaux (2018). Guide de Soumission d’une Demande à l’INESSS. INESSS (QC). https://www.inesss.qc.ca/fileadmin/doc/INESSS/Inscription_medicaments/Fiches_inscription/Guide_soumission.pdf.

[9] CADTH Database Search Filters (2021). Ottawa: CADTH. https://www.cadth.ca/strings-attached-cadths-database-search-filters.

[10] Covidence Systematic Review Software Veritas Health Innovation, Melbourne, Australia. www.covidence.org.

[11] Wijnen B., Van Mastrigt G., Redekop W.K., Majoie H., De Kinderen R., Evers S. (2016). How to prepare a systematic review of economic evaluations for informing evidence-based healthcare decisions: Data extraction, risk of bias, and transferability (part 3/3). Expert Rev. Pharm. Outcomes Res..

[12] Buckley L.F., Dixon D.L., Wohlford G.F., Wijesinghe D.S., Baker W.L., Van Tassell B.W. (2018). Effect of intensive blood pressure control in patients with type 2 diabetes mellitus over 9 years of follow-up: A subgroup analysis of high-risk ACCORDION trial participants. Diabetes Obes. Metab..

[13] Evers S., Goossens M., de Vet H., van Tulder M., Ament A. (2005). Criteria list for assessment of methodological quality of economic evaluations: Consensus on Health Economic Criteria. Int. J. Technol. Assess Health Care.

[14] Knies S., Ament A.J., Evers S.M., Severens J.L. (2009). The transferability of economic evaluations:testing the model of Welte. Value Health.

[15] Welte R., Feenstra T., Jager H., Leidl R. (2004). A decision chart for assessing and improving the transferability of economic evaluation results between countries. Pharmacoeconomics.

[16] (2022). The Professional Society for Health Economics and Outcomes Research: Pharmacoeconomic Guidelines Around The World. ISPOR. https://tools.ispor.org/peguidelines/.

[17] (2021). Candian Cancer Society: Prostate Cancer Statistics. https://cancer.ca/en/cancer-information/cancer-types/prostate/statistics.

[18] (2022). The World Bank: Life Expectancy at Birth, Total (Years). https://data.worldbank.org/indicator/SP.DYN.LE00.IN?locations=CN.

[19] (2020). International Agency for Cancer Research: Population Fact Sheets. World Health Organization. https://gco.iarc.fr/today/fact-sheets-populations.

[20] OFX Yearly Average Rates. https://www.ofx.com/en-ca/forex-news/historical-exchange-rates/yearly-average-rates/.

[21] Statistics Canada (2020). Consumer Price Index, Annual Average, Not Seasonally Adjusted.

[22] Page M.J., McKenzie J.E., Bossuyt P.M., Boutron I., Hoffmann T.C., Mulrow C.D., Shamseer L., Tetzlaff J.M., Akl E.A., Brennan S.E. (2021). The PRISMA 2020 statement: An updated guideline for reporting systematic reviews. BMJ.

[23] Pharmacoeconomic Report Apalutamide (Erleada) for Metastatic Castration-Sensitive Prostate Cancer. pERC Meeting: 20 March 2020; Early Conversion 22 April 2020; Toronto (ON) 2020. https://www.cadth.ca/sites/default/files/pcodr/Reviews2020/10200ApalutamidemCSPC_fnEGR_REDACT-ABBREV_EC_22Apr2020_final.pdf.

[24] Cadth Drug Reimbursement Review (2020). Pharmacoeconomic Report for Enzalutamide (XtandI) (Astellas Pharma Canada, Inc.) Indication: In Combination with Androgen-Deprivation Therapy for the Treatment of Patients with Metastatic Castration Sensitive Prostate Cancer. Toronto (ON): CADTH. https://www.cadth.ca/sites/default/files/pcodr/Reviews2020/10209EnzalutamidemCSPC_fnEGR_REDACT-ABBREV_Post_23Sep2020_final.pdf.

[25] Institut National D’excellence en Santé et en Services Sociaux (2020). ERLEADA MC—Cancer de la Prostate. Quebec (QC) INESSS. https://www.inesss.qc.ca/fileadmin/doc/INESSS/Inscription_medicaments/Avis_au_ministre/Juin_2020/Erleada_2020_05.pdf.

[26] Institut National D’excellence en Santé et en Services Sociaux (2020). XTANDI MC—Cancer de la Prostate Métastatique Sensible à la Castration. Quebec (QC) INESSS. https://www.inesss.qc.ca/fileadmin/doc/INESSS/Inscription_medicaments/Avis_au_ministre/Septembre_2020/Xtandi_CPSCm_2020_08.pdf.

[27] (2020). Scottish Medicines Consortium—SMC, Abiraterone Acetate (Zytiga). https://www.scottishmedicines.org.uk/medicines-advice/abiraterone-acetate-zytiga-full-smc2215/.

[28] National Institute for Health and Care Excellence Enzalutamide for Treating Hormone-Sensitive Metastatic Prostate Cancer—Guidance (TA712). NICE (UK) 2021. https://www.nice.org.uk/guidance/ta712.

[29] National Institute for Health and Care Excellence Abiraterone for Treating Newly Diagnosed High-Risk Hormone-Sensitive Metastatic Prostate Cancer—Guidance (TA721). NICE (UK) 2021. https://www.nice.org.uk/guidance/ta721.

[30] National Institute for Health and Care Excellence Apalutamide with Androgen Deprivation Therapy for Treating Hormone-Sensitive Metastatic Prostate Cancer—Guidance (TA741). NICE (UK) 2021. https://www.nice.org.uk/guidance/ta741.

[31] Aguiar P.N., Tan P.S., Simko S., Barreto C.M.N., Gutierres B.S., Giglio A.D., Lopes G.L. (2019). Cost-effectiveness analysis of abiraterone, docetaxel or placebo plus androgen deprivation therapy for hormone-sensitive advanced prostate cancer. Einstein.

[32] Beca J., Majeed H., Chan K.K.W., Hotte S.J., Loblaw A., Hoch J.S. (2019). Cost-effectiveness of docetaxel in high-volume hormone-sensitive metastatic prostate cancer. Can. Urol. Assoc. J..

[33] Parikh N.R., Chang E.M., Nickols N.G., Rettig M.B., Raldow A.C., Steinberg M.L., Koontz B.F., Vapiwala N., Deville C., Feng F.Y. (2020). Cost-Effectiveness of Metastasis-Directed Therapy in Oligorecurrent Hormone-Sensitive Prostate Cancer. Int. J. Radiat. Oncol. Biol. Phys..

[34] Pelloux-Prayer R., Schiele P., Oudard S., Gravis G., Kleinclauss F., Crehange G., Hennequin C., Morgans A., Geoffrois L., Limat S. (2021). Cost-effectiveness Analysis of Innovative Therapy for Patients with Newly Diagnosed Hormone-Sensitive Metastatic Prostate Cancer. Clin. Genitourin. Cancer.

[35] Ramamurthy C., Handorf E., Correa A., Beck J., Geynisman D. (2019). Cost-effectiveness of abiraterone versus docetaxel in the treatment of metastatic hormone naive prostate cancer. Urol. Oncol..

[36] Sathianathen N., Alarid-Escudero F., Kuntz K., Lawrentschuk N., Bolton D., Murphy D., Kim S., Konety B. (2019). A Cost-effectiveness Analysis of Systemic Therapy for Metastatic Hormone-sensitive Prostate Cancer. Eur. Urol. Oncol..

[37] Woods B., Sideris E., Sydes M., Gannon M., Parmar M., Alzouebi M., Attard G., Birtle A., Brock S., Cathomas R. (2018). Addition of Docetaxel to First-line Long-term Hormone Therapy in Prostate Cancer (STAMPEDE): Modelling to Estimate Long-term Survival, Quality-adjusted Survival, and Cost-effectiveness. Eur. Urol. Oncol..

[38] Zhang P., Wen F., Fu P., Yang Y., Li Q. (2017). Addition of docetaxel and/or zoledronic acid to standard of care for hormone-naive prostate cancer: A cost-effectiveness analysis. Tumori.

[39] Zhang P., Xie D., Li Q. (2021). Adding Enzalutamide to First-Line Treatment for Metastatic Hormone-Sensitive Prostate Cancer: A Cost-Effectiveness Analysis. Front. Public Health.

[40] Zheng H.R., Wen F., Wu Y.F., Wheeler J.R.C., Li Q. (2017). Cost-effectiveness analysis of additional docetaxel for metastatic hormone-sensitive prostate cancer treated with androgen-deprivation therapy from a Chinese perspective. Eur. J. Cancer Care.

[41] Aguiar P., Barreto C., Gutierres B., Tadokoro H., Lopes G. (2017). Cost effectiveness of chemohormonal therapy in patients with metastatic hormone-sensitive and non-metastatic high-risk prostate cancer. Einstein.

[42] National Institute for Health and Care Excellence Hormone-Sensitive Metastatic Prostate Cancer: Docetaxel—Evidence Summary (ESUOM50). NICE (UK) 2016. https://www.nice.org.uk/advice/esuom50.

[43] pCODR Final Economic Guidance Report—Darolutamide (Nubeqa) for Non-Metastatic Castration Resistant Prostate Cancer. pERC Meeting: 19 March 2020; Early Conversion: 22 April 2020; Unredacted: 5 February 2021; Toronto (ON) 2021. https://www.cadth.ca/sites/default/files/pcodr/Reviews2020/10196DarolutamidenmCRPC_fnEGR_REDACT-ABBREV_EC_22Apr2020_final.pdf.

[44] pCODR Final Economic Guidance Report—Apalutamide (Erleada) for Castration-Resistant Prostate Cancer. pERC Meeting: 16 August 2018; pERC Reconsideration Meeting: 18 October 2018. Toronto (ON) 2018. https://www.cadth.ca/sites/default/files/pcodr/pcodr_apalutamide_erleada_crpc_fn_egr.pdf.

[45] (2019). pan-Canadian Oncology Drug Review Final Economic Guidance Report, Enzalutamide (Xtandi) for Non-Metastatic Castration-Resistant Prostate Cancer. Toronto (ON): CADTH. https://cadth.ca/sites/default/files/pcodr/Reviews2019/10149Enzalutamidenm-CRPC_fnEGR_EC_NOREDACT-ABBREV_Post_26Mar2019_final.pdf.

[46] Institut National D’excellence en Santé et en Services Sociaux (2020). Nubequa MC—Cancer de la Prostate. Quebec (QC) INESSS. https://www.inesss.qc.ca/fileadmin/doc/INESSS/Inscription_medicaments/Avis_au_ministre/Avril_2020/Nubeqa_2020_03.pdf.

[47] Institut National D’excellence en Santé et en Services Sociaux (2018). Erleada MC—Cancer de la Prostate. Quebec (QC) INESSS. https://www.inesss.qc.ca/fileadmin/doc/INESSS/Inscription_medicaments/Avis_au_ministre/Octobre_2018/Erleada_2018_09.pdf.

[48] National Institute for Health and Care Excellence Darolutamide with Androgen Deprivation Therapy for Treating Hormone-relapsed Non-Metastatic Prostate Cancer—Guidance (TA660). NICE (UK) 2020. https://www.nice.org.uk/guidance/ta660.

[49] National Institute for Health and Care Excellence Enzalutamide for Hormone-Relapsed Non-Metastatic Prostate Cancer—Guidance (TA580). NICE (UK) 2019. https://www.nice.org.uk/guidance/ta580.

[50] Scottish Medicines Consortium Darolutamide (Nubeqa) is Accepted for Use within NHS Scotland. SMC (SC) 2020. https://www.scottishmedicines.org.uk/medicines-advice/darolutamide-nubeqa-full-smc2297/.

[51] Scottish Medicines Consortium Enzalutamide 40 mg Soft Capsules (Xtandi®). SMC (UK) 2019. https://www.scottishmedicines.org.uk/medicines-advice/enzalutamide-xtandi-full-smc2195/.

[52] National Institute for Health and Care Excellence Apalutamide with Androgen Deprivation Therapy for Treating High-Risk Hormone-Relapsed Non-Metastatic Prostate Cancer—Guidance (TA740). NICE (UK) 2021. https://www.nice.org.uk/guidance/ta740.

[53] Toro W., Braun S., Sanchez L., Anaya P. (2020). Pcn129 a Cost-Utility and Budget Impact Analysis of Enzalutamide for the Treatment of Nonmetastatic Castration-Resistant Prostate Cancer (Nmcrpc) in Mexico. Value Health.

[54] Tsiatas M., Van Oostrum I., Tritaki G., Sermon J., Chatzimouratidis K. (2019). Pcn218 Cost-Effectiveness of Apalutamide + Adt Versus Enzalutamide + Adt in Non-Metastatic Castration Resistant Prostate Cancer in Greece. Value Health.

[55] Zhou Z., Hu X. (2018). Cost-Effectiveness Analysis of Apalutamide for Treatment in Non- Metastasis Castration-Resistant Prostate Cancer. Value Health.

[56] Hu X., Qu S., Yao X., Li C., Liu Y., Wang J. (2019). Abiraterone acetate and docetaxel with androgen deprivation therapy in high-volume metastatic hormone-sensitive prostate cancer in China: An indirect treatment comparison and cost analysis. Cost Eff. Resour. Alloc..

[57] Ke X., Lafeuille M., Romdhani H., Kinkead F., Pilon D., Lefebvre P., Francis P., D’Andrea D., Ryan C., Freedland S. (2019). Healthcare resource use and costs associated with metastatic castration-sensitive prostate cancer in medicare advantage and commercially insured patients in The United States. J. Manag. Care Spec. Pharm..

[58] Wong S., Everest L., Jiang D., Saluja R., Chan K., Sridhar S. (2020). Application of the ASCO Value framework and ESMO magnitude of clinical benefit scale to assess the value of abiraterone and enzalutamide in advanced prostate cancer. JCO Oncol. Pract..

[59] Svensson J., Lissbrant I., Gauffin O., Hjalm-Eriksson M., Kilany S., Fagerlund K., Stattin P. (2021). Time spent in hormone-sensitive and castration-resistant disease states in men with advanced prostate cancer, and its health economic impact: Registry-based study in Sweden. Scand. J. Urol..

[60] Freedland S., Pilon D., Bhak R., Lefebvre P., Li S., Young-Xu Y. (2020). Predictors of survival, healthcare resource utilization, and healthcare costs in veterans with non-metastatic castration-resistant prostate cancer. Urol. Oncol. Semin. Orig. Investig..

[61] George D., Schultz N., Huang A., Wang L., Baser O., Ramaswamy K., Mardekian J. (2018). Increased costs associated with progression to metastatic castrate-resistant prostate cancer. J. Manag. Care Spec. Pharm..

[62] Seal B., Sullivan S., Ramsey S., Asche C., Shermock K., Sarma S., Zagadailov E., Farrelly E., Eaddy M. (2014). Comparing hospital-based resource utilization and costs for prostate cancer patients with and without bone metastases. Appl. Health Econ. Health Policy.

[63] Shah A., Shah R., Kebede N., Mohamed A., Botteman M., Waldeck R., Hussain A. (2020). Real-world incidence and burden of adverse events among non-metastatic prostate cancer patients treated with secondary hormonal therapies following androgen deprivation therapy. J. Med. Econ..

[64] Wu B., Li S., Song J., Pericone C., Behl A., Dawson N. (2020). Total cost of care for castration-resistant prostate cancer in a commercially insured population and a medicare supplemental insured population. J. Med. Econ..

[65] Zhang P., Xie D., Li Q. (2021). Cost-effectiveness analysis of cabazitaxel for metastatic castration resistant prostate cancer after docetaxel and androgen-signaling-targeted inhibitor resistance. BMC Cancer.

[66] Zhang A.Y., Fu A.Z. (2016). Cost-effectiveness of a behavioral intervention for persistent urinary incontinence in prostate cancer patients. Psycho-Oncology.

[67] Medicare Advantage Plans. https://www.medicare.gov/sign-up-change-plans/types-of-medicare-health-plans/medicare-advantage-plans.

[68] Medicare Advantage vs. Medigap. https://www.investopedia.com/articles/personal-finance/071014/medigap-vs-medicare-advantage-which-better.asp.

[69] (2017). Guidelines for the Economic Evaluation of Health Technologies: Canada.

[70] The pan-Canadian Pharmaceutical Alliance. https://www.pcpacanada.ca/node/30.

[71] The pan-Canadian Pharmaceutical Alliance Brand Name Drug Negotiations Status. https://www.pcpacanada.ca/negotiations.

[72] Régie de L’assurance Maladie Quebec Liste des Médicaments. RAQM (QC) 2 March 2022. https://www.ramq.gouv.qc.ca/sites/default/files/documents/liste_med_2022-03-02_fr.pdf.

[73] Fizazi K., Shore N., Tammela T.L., Ulys A., Vjaters E., Polyakov S., Jievaltas M., Luz M., Alekseev B., Kuss I. (2020). Nonmetastatic, Castration-Resistant Prostate Cancer and Survival with Darolutamide. N. Engl. J. Med..

[74] Small E.J., Saad F., Chowdhury S., Oudard S., Hadaschik B.A., Graff J.N., Olmos D., Mainwaring P.N., Lee J.Y., Uemura H. (2019). Apalutamide and overall survival in non-metastatic castration-resistant prostate cancer. Ann. Oncol..

[75] Sternberg C.N., Fizazi K., Saad F., Shore N.D., De Giorgi U., Penson D.F., Ferreira U., Efstathiou E., Madziarska K., Kolinsky M.P. (2020). Enzalutamide and Survival in Nonmetastatic, Castration-Resistant Prostate Cancer. N. Engl. J. Med..

[76] Régie de L’assurance Maladie du Québec Liste des Médicaments. Québec (QC); 13 December 2021. https://www.ramq.gouv.qc.ca/sites/default/files/documents/liste_med_2021-12-15_fr.pdf.

[77] Fujiwara M., Yuasa T., Komai Y., Numao N., Yamamoto S., Fukui I., Yonese J. (2020). Efficacy, Prognostic Factors, and Safety Profile of Enzalutamide for Non-metastatic and Metastatic Castration-Resistant Prostate Cancer: A Retrospective Single-Center Analysis in Japan. Target Oncol..

[78] Jaime Caro J., Eddy D.M., Kan H., Kaltz C., Patel B., Eldessouki R., Briggs A.H. (2014). Questionnaire to Assess Relevance and Credibility of Modeling Studies for Informing Health Care Decision Making: An ISPOR-AMCP-NPC Good Practice Task Force Report. Value Health.

[79] Philips Z., Bojke L., Sculpher M., Claxton K., Golder S. (2006). Good Practice Guidelines for Decision-Analytic Modelling in Health Technology Assessment. PharmacoEconomics.

[80] Grochtdreis T., König H.-H., Dobruschkin A., Von Amsberg G., Dams J. (2018). Cost-effectiveness analyses and cost analyses in castration-resistant prostate cancer: A systematic review. PLoS ONE.

[81] Grover S.A., Zowall H., Coupal L., Krahn M. (1999). Prostate cancer: 12. The economic burden. CMAJ Can. Med. Assoc. J..

